# A Gene Family Derived from Transposable Elements during Early Angiosperm Evolution Has Reproductive Fitness Benefits in *Arabidopsis thaliana*

**DOI:** 10.1371/journal.pgen.1002931

**Published:** 2012-09-06

**Authors:** Zoé Joly-Lopez, Ewa Forczek, Douglas R. Hoen, Nikoleta Juretic, Thomas E. Bureau

**Affiliations:** Department of Biology, McGill University, Montreal, Quebec, Canada; University of Georgia, United States of America

## Abstract

The benefits of ever-growing numbers of sequenced eukaryotic genomes will not be fully realized until we learn to decipher vast stretches of noncoding DNA, largely composed of transposable elements. Transposable elements persist through self-replication, but some genes once encoded by transposable elements have, through a process called molecular domestication, evolved new functions that increase fitness. Although they have conferred numerous adaptations, the number of such domesticated transposable element genes remains unknown, so their evolutionary and functional impact cannot be fully assessed. Systematic searches that exploit genomic signatures of natural selection have been employed to identify potential domesticated genes, but their predictions have yet to be experimentally verified. To this end, we investigated a family of domesticated genes called *MUSTANG* (*MUG*), identified in a previous bioinformatic search of plant genomes. We show that *MUG* genes are functional. Mutants of *Arabidopsis thaliana MUG* genes yield phenotypes with severely reduced plant fitness through decreased plant size, delayed flowering, abnormal development of floral organs, and markedly reduced fertility. *MUG* genes are present in all flowering plants, but not in any non-flowering plant lineages, such as gymnosperms, suggesting that the molecular domestication of *MUG* may have been an integral part of early angiosperm evolution. This study shows that systematic searches can be successful at identifying functional genetic elements in noncoding regions and demonstrates how to combine systematic searches with reverse genetics in a fruitful way to decipher eukaryotic genomes.

## Author Summary

The genomes of complex organisms are mostly made up not of ordinary genes but of transposable elements. Transposable elements have been called “selfish DNA” because they normally persist by copying themselves, not by helping the organism to survive or reproduce. Yet transposable elements can help organisms to evolve; for instance, transposable element genes sometimes acquire new functions that do benefit the organism. Because they are difficult to distinguish from transposable elements, little is known about these “domesticated genes.” Although studies have attempted to identify them computationally, the predictions have not been verified experimentally. Here, we examine some of the first domesticated genes to be predicted computationally, the *MUSTANG* family of plant genes. We show that the predictions were correct: *MUSTANGs* are, like ordinary genes, functional. MUSTANG mutations result in serious defects in how plants grow, flower, and reproduce. Since they are present only in flowering plants, *MUSTANG* probably originated when flowers first evolved, perhaps taking on a key role. This study is important both because it shows that *MUSTANG* is critical to plant fitness and because, in the future, a similar approach can be used to find additional domesticated genes and to better understand how transposable elements contribute to evolution.

## Introduction

Recent technological advances have enabled the sequencing of hundreds of eukaryotic genomes, emphasizing that protein-coding genes constitute only a small fraction of the DNA [1], [2]. Since then, attention has increasingly shifted to deciphering other parts of the genome, the so-called non-coding regions that are largely composed of transposable elements (TEs). Unlike canonical genes, TEs can persist without benefiting fitness by replicating through transposition within the genome [3], [4]. There are two major classes of TEs, which transpose by fundamentally different mechanisms: retrotransposons, by reverse transcription of an RNA intermediate; and DNA transposons, by cut and paste transposition. TEs in each active family encode the proteins they need for transposition, which differ by replication strategy and superfamily [5].

Although traditionally viewed as selfish [3], [4], evidence continues to mount that TEs affect how genomes evolve in a variety of beneficial ways. One example is molecular domestication, where TEs are repurposed into new genes or other sequences with novel functions [6]–[10]. TE genes are adapted for transposition-related functions, yet they have molecular properties, such as DNA binding and protein-protein interaction, that can be used as raw genetic material and co-opted to perform new functions. Domesticated TEs (DTEs) are different from TEs and similar to canonical genes both in terms of their structure and activity, and in that they are subject to phenotypic selection. Most known DTEs have co-linear orthologs and have lost transposition-related features such as flanking terminal repeats and transposase catalytic activity [8], [10]. However, other features of the original TE that contribute to the beneficial function are maintained, such as conserved domains. DTEs perform various beneficial functions; for example, many DTEs derived from DNA transposons are transcription factors [7], while others are involved in centromere binding, chromosome segregation, meiotic recombination, heterochromatin formation, TE silencing, programmed genome rearrangement, V(D)J recombination, genome stability, and translational regulation [8], [11]. Molecular domestication has helped to spur remarkable evolutionary innovations, including the mammalian placenta and the vertebrate adaptive immune system [8].

To date, most DTEs have been discovered fortuitously by forward genetics. In plants for example, the *FHY3* (*FAR-RED ELONGATED HYPOCOTYL 3*) family and *DAYSLEEPER* are the only well-characterized DTEs. *FHY3* and *FAR1* (*FAR-RED IMPAIRED RESPONSE 1*), two members of the *FHY3* family, were identified in screens for far-red light mutants [12]–[14]. *DAYSLEEPER*, which is essential to plant development, was identified in a yeast one-hybrid screen [15]. Even if only a small fraction of the tens of thousands of TE-like genes in plant genome are DTEs rather than TEs, the total number of DTEs may be much higher than currently reported, suggesting that many more may await discovery and that traditional genetic methods may be insufficient to find them, for example due to functional redundancy.

These limitations can be overcome by direct bioinformatic searches of genomic data in which DTE genes are discriminated from TEs using genomic signatures that result from differences between how TEs and DTEs function and evolve, such as differences in expression, microsynteny, evolutionary rate, phylogeny, repetitiveness, and TE termini [2], [16]–[21]. But while theoretically sound, bioinformatics-based searches for DTE genes have not yet been confirmed experimentally. To assess the validity of this approach, we investigated a family of DTEs, *MUSTANG* (*MUG*), identified in a previous bioinformatic search of plant genomes [17], using a reverse genetic approach. *MUG* sequences are similar to ancestral TEs called *Mutator*-like elements (MULEs), but unlike MULEs, *MUG* genes lack signature terminal sequences, are collinear in monocots and eudicots, are functionally constrained, and are differentially expressed [17], [22], [23]. Here, we show that *MUG* genes have been conserved throughout angiosperm evolution and experimentally validate that they are functional in *Arabidopsis thaliana* by showing that they are essential to flower development and plant fitness.

## Results

### *MUG* subfamilies are conserved among angiosperms

**Figure 1:**
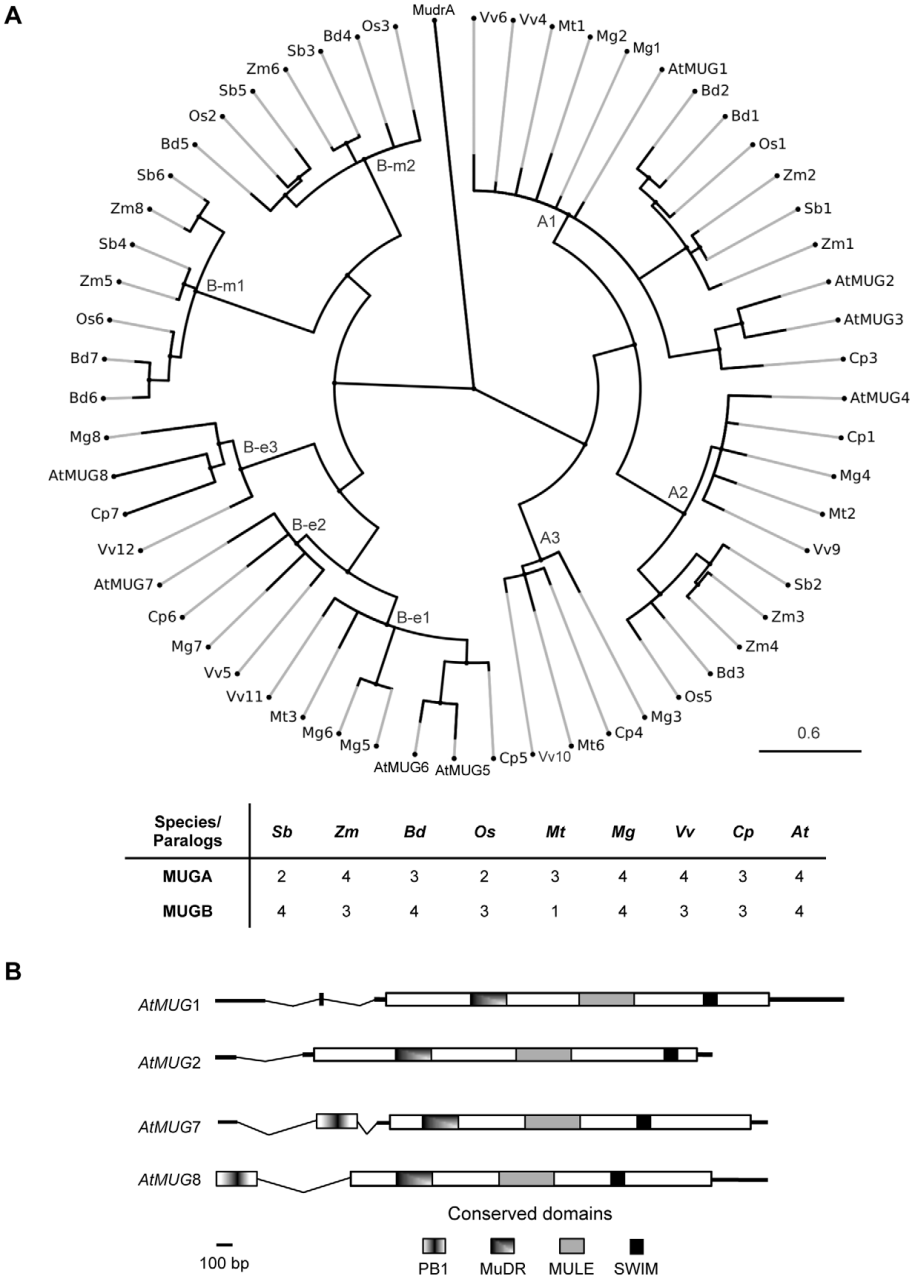
*MUSTANG* phylogeny and gene structure in *A. thaliana*. (A) *MUG* phylogeny in nine angiosperm species. At, *Arabidopsis thaliana*; Bd, *Brachypodium distachyon*; Cp, *Carica papaya*; Mg, *Mimulus guttatus*; Mt, *Medicago truncatula*; Os, *Oryza sativa*; Sb, *Sorghum bicolor*; Vv, *Vitis vinifera*; Zm, *Zea mays*. See Figure S1 for sequences and Table S5 for locus IDs. All bootstrap values are >70% (not shown). Cp7 is truncated; its position is approximate. (B) Graphical representation of *At-MUG1*, *At-MUG2*, *At-MUG7*, and *At-MUG8* gene transcripts. Bold horizontal lines represent transcripts, dips introns, and rectangles coding sequences.

To investigate the size and distribution of the *MUG* gene family across a wide range of taxa, we searched for complete sets of *MUG* paralogs in nine angiosperm species with high quality full-genome sequencing projects at an advanced stage or completed (Figure S1). Phylogenetic analysis revealed that *MUG* consists of two major subfamilies, *MUGA* and *MUGB*, both present in all nine species, but with different patterns of lineage-specific diversification (Figure 1, Figure S2). We found a minimum of four and a maximum of eight *MUG* genes per species, including the eight previously identified in *A. thaliana* [17], which we call *At-MUG1* to *At-MUG8*. There may be additional *MUG* genes in unassembled parts of the genomes that we could not identify. These results are consistent with previous analyses of sugarcane (*Saccarum officinarum* and *Saccarum spontaneum*) [19], [24], which found 5 *MUGA* and 10 *MUGB* genes, referred to as “Class III” and “Class IV”, respectively, and of grapevine (*Vitis vinifera*) [21], which found 5 *MUGA* (we found only 4) and 3 *MUGB* genes, referred to as MUGvine1–5 and MUGvine6–8, respectively.

Consistent with previous reports showing that *MUG* genes were present prior to the monocot-eudicot split [17], [19], [24], we found that the *MUGA* subfamily diversified prior to the divergence of monocots and eudicots into three extant clades, A1 to A3. Clade A1 has at least one member in each examined species and includes *At-MUG1*, *At-MUG2*, and *At-MUG3*, the latter two belonging to a Brassicales-specific subclade. Clade A2, which includes *At-MUG4*, has exactly one copy in every species, except *Zea mays*, in which it appears to have a recent duplication. Clade A3 differs from A1 and A2. Although it was the first of these three clades to diverge, apparently prior to the monocot-eudicot split, it has no member in the examined monocot species, nor is there a clade A3 member in *A. thaliana*.

Conversely, the diversification of the *MUGB* subfamily did not occur until after the monocot-eudicot split, into two monocot-specific clades (B-m1 and B-m2) and three eudicot-specific clades (B-e1 to B-e3). Each examined monocot species has one or two members of each monocot-specific clade. The branches leading to these clades are long, suggesting that B-m1 and B-m2 may have diverged early in monocot evolution. Similarly, all examined eudicots have one or two members in the eudicot-specific clade B-e1, which includes *At-MUG5* and *At-MUG6*. Clades B-e2, which includes *At-MUG7*, and B-e3, which includes *At-MUG8*, each consist of exactly one member from each examined eudicot species, except *Medicago truncatula*, which has no member in either B-e2 or B-e3. Clade B-e3 is the most divergent *MUG* clade. Together, these results suggest that most *MUG* clades are conserved within lineages, with *MUGA* clades encompassing both monocots and eudicots and *MUGB* clades specific to monocots or eudicots.

To further investigate the origin of the *MUG* family and its distribution among plant taxa, we searched expressed sequence tag (EST) libraries deposited at the Ancestral Angiosperm Genome Project (AAGP; http://ancangio.uga.edu) and the National Center for Biotechnology Information (NCBI) dbEST [25]. We identified multiple ESTs similar to *A. thaliana MUG* genes in all five ancestral angiosperm species represented in the AAGP (Table S1), as well as in every order of angiosperm in dbEST with a sufficiently large library (5,000 ESTs or more), except Acorales, Alismatales, and Liliales (Table S2). No putative *MUG* ESTs were found in any gymnosperm in the AAGP or dbEST, nor in any other taxa outside angiosperms, although putative MULEs were found in all taxa. Consistent with these EST results, the sequences most similar to *MUG* in the genomes of *Selaginella moellendorffii* (a primitive vascular plant) and *Physcomitrella patens* (a moss) are repetitive and contain premature stop codons, characteristics indicative of TEs. Phylogenetic analyses of aligned selections of ESTs and genomic sequences confirmed these results (data not shown).

All *MUG* genes contain three conserved domains, an N-terminal MuDR DNA-binding domain (Pfam PF03108) [26], a core MULE transposase domain (PF10551), and a C-terminal SWIM zinc-finger domain (PF04434) (Figure 1B, Figure S2). The same domain architecture is found in diverse transposases of the MULE superfamily [27]. *MUGA* and *MUGB* members encode all three domains in a single exon. In addition, *MUGB* genes have an additional short 5′ exon encoding the Phox and Bem1p (PB1) domain (PF00564).

*MUGA* and *MUGB* subfamilies have synonymous nucleotide substitution rates (dN/dS) of 0.12 and 0.10, respectively, which are significantly less than one (p = 0.0045 and p = 0.0085, respectively), showing that they are under purifying selection.

### Creation of *MUG* double mutants for reverse genetics

To test for *MUG* functionality in *A. thaliana*, we used a reverse genetics approach. Two independently derived mutant alleles with T-DNA insertions into the coding regions were obtained from the Arabidopsis Biological Research Center, Ohio State University. When grown under standard conditions, homozygous single mutants show no obvious differences from wild-type (data not shown). This suggests that *MUG* genes may be functionally redundant, as is often observed in plant gene families. To test this, we crossed single mutants within each subfamily to obtain homozygous double mutants. The *mug1 mug2* (*MUGA* subfamily) and *mug7 mug8* (*MUGB* subfamily) double mutants exhibit strong phenotypes, consistent with the hypothesis that *MUG1* has some degree of functional redundancy with *MUG2*, and *MUG7* with *MUG8*.

### *MUG* double mutants have severe developmental and reproductive defects

**Figure 2:**
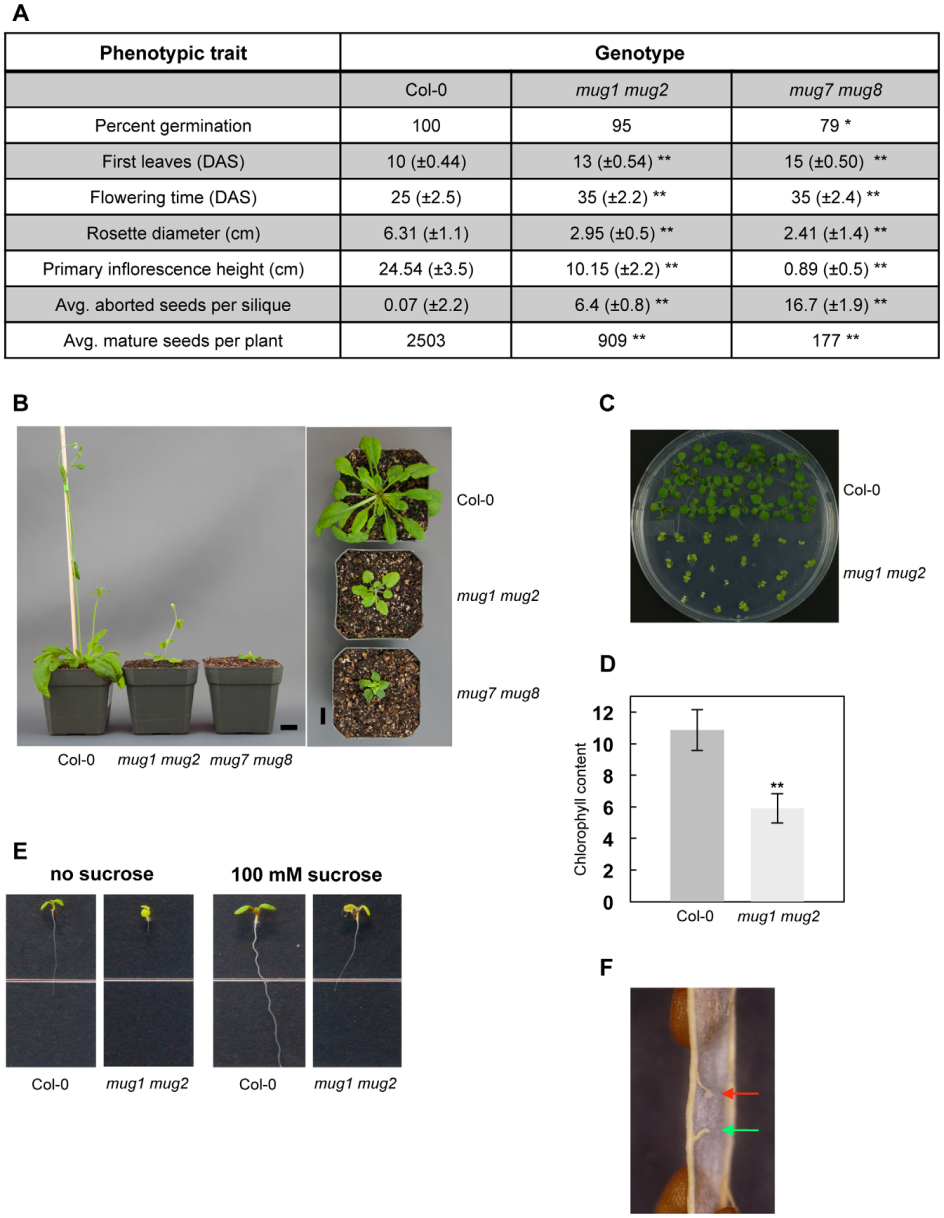
Phenotypic analysis of *mug1 mug2* and *mug7 mug8* in *A. thaliana*. (A) Phenotypes of wild-type (Col-0), *mug1 mug2*, and *mug7 mug8* based on the following traits: 1) Germination (%); 2) First leaves recorded as number of days after sterilization (DAS); 3) Flowering time (DAS); 4) Rosette leaf coloration; 5) Rosette diameter (cm) at 42 DAS; 6) Height (cm) of primary inflorescence at 42 DAS; 7) Average number of aborted seeds. Measurements based on 60 plants per genotype. Statistical significance based on two-sample student *t*-test; α = 0.05; * p<0.01; ** p<0.001. (B) Growth phenotype of Col-0, *mug1 mug2*, and *mug7 mug8* at 40 DAS. Scale = 1 cm. (C) Image of an MS plate (0.8% agar w/v; 1% sucrose w/v) containing 17-day-old Col-0 and *mug1 mug2* seedlings. (D) Chlorophyll accumulation in Col-0 and *mug1 mug2* seedlings. Bars represents standard deviations of quadruple experiments. 200 mg or approximately 10 Col-0 or 30 *mug1 mug2* seedlings per experiment. (E) Phenotypes of *mug1 mug2* under sucrose conditions. Twelve-day-old seedlings of Col-0 and *mug1 mug2* were grown on standard MS medium without sucrose or with 100 mM sucrose. (F) Close up of a dissected *mug1 mug2* silique showing a normal funiculus from which the attached mature seed was released (green arrow) and a funiculus attached to undeveloped ovule tissues (red arrow).

To characterize the double mutant phenotypes, we measured a number of traits that reflect plant fitness at various life stages (Table S3). We compared two different allelic combinations each of *mug1 mug2* and *mug7 mug8* mutants to wild-type *A. thaliana* plants, ecotype Col-0 (Figure S3). When grown under standard laboratory conditions, the double mutants differ dramatically from wild-type at all developmental stages from germination to senescence. They exhibit phenotypes that include reduced plant size, an increased incidence of aborted seeds, reduced seed amount, and delays in developmental timing and flowering (Figure 2A). Although the two double mutants have defects in several similar traits, the degree of severity for some traits is stronger in *mug7 mug8* than in *mug1 mug2*. *mug1 mug2* yields 36% of the wild-type seed set, whereas *mug7 mug8* yields only 7%. *mug1 mug2* stems are more than 2 times shorter than wild-type, whereas those of *mug7 mug8* are more than 27 times shorter. Interestingly, *mug1 mug2* has a ratio of stem height to rosette diameter (r = 3.44) similar to wild-type (r = 3.89), whereas *mug7 mug8* has a much-reduced ratio (r = 0.37) (Figure 2B). Application of exogenous gibberellic acid (GA) failed to rescue reduced stem height or delayed flowering phenotypes (data not shown). The growth defect seems to be restricted to light conditions, since both double mutants show hypocotyls that etiolate normally (data not shown).

Some traits are unique to each double mutant. *mug7 mug8* leaves are slightly curly, whereas *mug1 mug2* leaves have a pale yellow-green coloration, which is most evident in seedlings but remains visible in mature plants (Figure 2B, 2C). *mug1 mug2* seedlings contain only about half the chlorophyll per unit mass of wild-type seedlings (Figure 2D). They also fail to develop beyond the cotyledon stage in the absence of exogenous sucrose, and even with exogenous sucrose their growth is restricted (Figure 2E).

**Figure 3:**
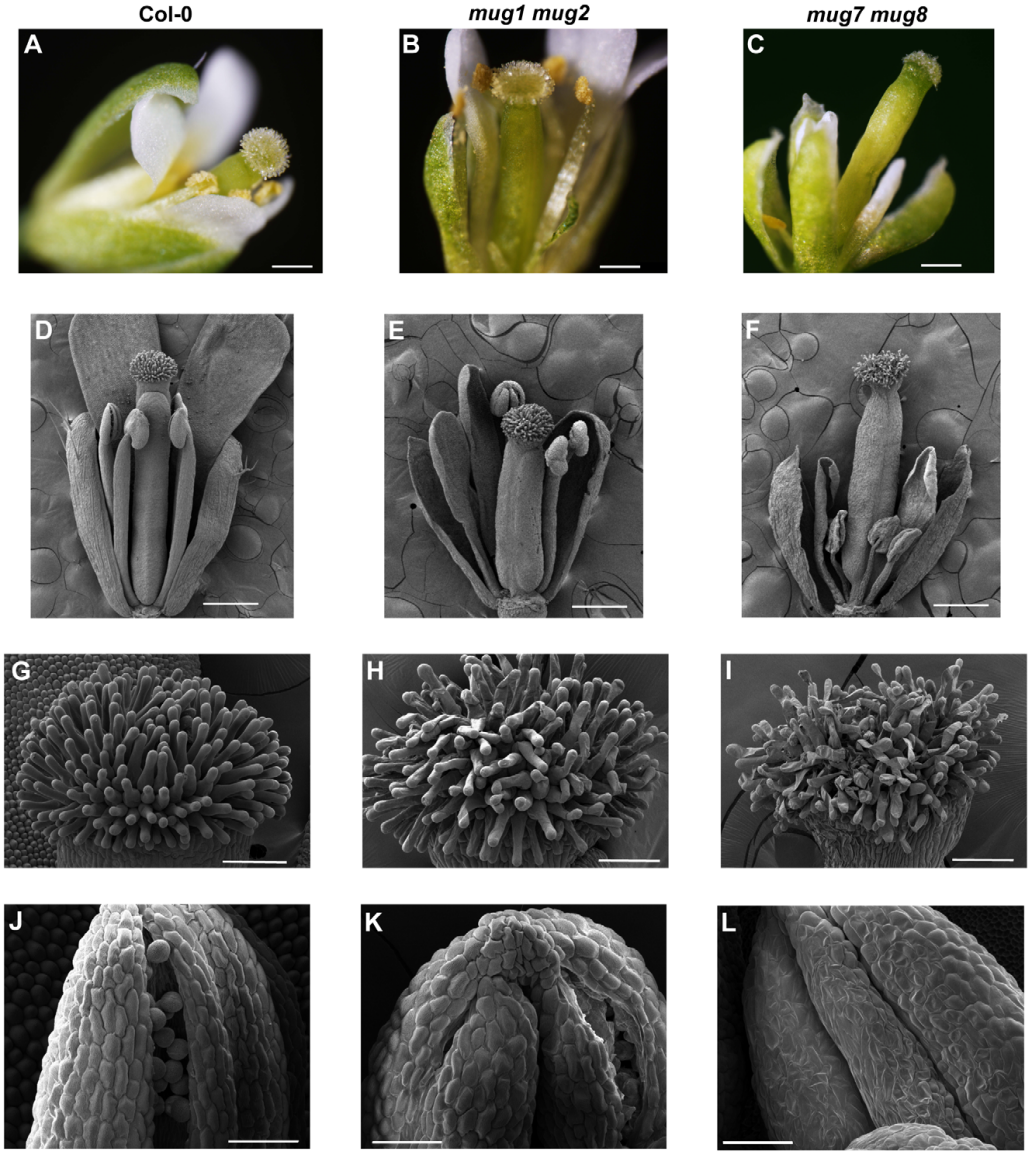
Flower structure of wild-type, *mug1 mug2*, and *mug7 mug8* in *A. thaliana*. (A–C) Bright-field micrographs of dissected flowers shown of (A) wild-type, (B) *mug1 mug2*, and (C) *mug7 mug8*. Scale = 0.5 mm. (D–F) SEM micrographs of dissected flowers of (D) wild-type, (E) *mug1 mug2*, and (F) *mug7 mug8*. Scale = 0.5 mm. (G–I) SEM micrographs of dissected flowers at higher magnification showing the stigma papillae of (G) wild-type, (H) *mug1 mug2*, and (I) *mug7 mug8*. Scale = 100 µm. (J–L) SEM micrographs of dissected flowers at higher magnification showing one anther and pollen of (J) wild-type, (K) *mug1 mug2*, and (L) *mug7 mug8*. Scale = 50 µm.

Each double mutant has defects in floral development and organ morphology that result in reduced fertility (Figure 3). The flowers are smaller than wild-type. Floral abnormalities are most dramatic at anthesis, especially in *mug7 mug8* (Figure 3). The gynoecium becomes highly elongated relative to the anther filaments (Figure 3C). Scanning electron microscopy (SEM) showed that the surface cell layers of certain flower organs, including the stigmatic hair-bearing region and the anthers, are deflated and have abnormal shapes, giving the flowers a shriveled appearance (Figure 3F). These observations suggest that *mug7 mug8* flowers may undergo premature senescence. Anthers are bilocular structures that normally produce and hold pollen grains, and upon maturity dehisce longitudinally to release the pollen. Most *mug7 mug8* anthers are flat and contain no pollen, and even when they do contain pollen, the locules usually fail to furrow and dehisce, preventing the pollen from being released (Figure 3L). The female organs of *mug7 mug8* are also defective, since applying wild-type pollen onto the mutant stigma did not rescue the fertility. *mug1 mug2* has less severe defects (Figure 3). The flowers are not shriveled and both the female and male organs are functional and capable of participating in self-fertilization. However, the anthers do exhibit restricted dehiscence, with only one of the two furrows opening successfully (Figure 3K). The siliques produced by both double mutants have a smaller maximum size than wild-type, restricting the number of seeds they bear, and they have a high incidence of undeveloped ovules, attenuating the seed yield (Figure 2A, 2F).

## Discussion

*In silico* screens like that in which the *MUG* gene family was identified [17] have the potential to detect many novel DTEs, but to be convincing their predictions must be validated experimentally. To do so, we exploited a fundamental difference between TE genes and canonical genes. Although TEs can produce phenotypes, for instance by inserting into and disabling a canonical gene, they are not known to themselves encode beneficial functions [11]. Therefore, knocking out a TE gene should have either no effect on fitness or should increase it, whereas knocking out a canonical gene, such as a DTE, may reduce the fitness of the organism. We utilized this difference by examining traits closely tied to fitness in *mug* T-DNA insertion mutants.

Fitness is a measure of how much a genotype contributes to the next generation in a given environment and includes components of survival and reproduction [28]. Both of these aspects are compromised in *mug* mutants, and certain phenotypes are especially striking. First, the survival of *mug* mutants is compromised by defects in their physiology (Figure 2, Figure 3). *mug7 mug8* plants are severe dwarfs, which may disadvantage them in various environments, such as where space or light is limited. The size of *mug1 mug2* plants is less reduced, but they have other serious defects. They contain only approximately half of the wild-type amount of chlorophyll per unit mass and their roots barely elongate in the absence of exogenous sucrose (Figure 2C–2E). Sucrose, the major product of photosynthesis, plays a key role in sugar signaling pathways and is required to supply metabolic energy to the roots [29]. Reduced chlorophyll concentrations could impair chloroplast activity and sucrose production, so may be one explanation of why *mug1 mug2* plants require exogenous sucrose, without which they would be unlikely to survive to maturity in wild environments.

Second, the reproduction of *mug* mutants is compromised by defects in the floral organ development, fecundity, and reproductive timing (Figure 2A, Figure 3). These defects are particularly severe in *mug7 mug8* mutants. They produce little or no pollen, their stamens and pistil do not elongate normally, preventing contact and limiting self-pollination, and their gynoecium is defective, limiting fertilization even if pollen does adhere to the stigmatic papillae (Figure 3). This combination of defects renders *mug7 mug8* mutants nearly sterile, with an average seed yield of only 7% of wild-type, even under optimal laboratory conditions.

The observed phenotypes may result from defects in a variety of physiological processes, so the function of MUG is not yet clear; however, patterns of conservation and expression do provide a few clues. Multiple lines of evidence, in addition to the mutant phenotypes, show that it is highly unlikely that *MUG* genes function in transposition. They lack the TE termini required for mobilization and are collinear in multiple genomes [17]; Most lack intact DDE motifs, which are required to catalyze transposition (Figure S2, Table S4) [27], [30]; Searches of publicly available data show that, unlike TEs [31], they are not targeted by small RNAs or silenced by DNA methylation [32]–[34], but are instead expressed in diverse tissues in *A. thaliana*, sugarcane [19], [24], [35], rice [36], and other angiosperms (Table S1, Table S2). The only other known MULE-like DTEs, the *FHY3* family, are transcription factors [30], [37], [38], a common function among domesticated DNA transposons [8], [11]. Like *FHY3*, the WRKY-GCM1, MULE, and SWIM domains of *MUG* genes are highly conserved, including key active site residues (Figure S2), suggesting that they may also have a function involving DNA binding, such as transcription regulation.

The severe defects and reduced fitness associated with mutations to *MUG* genes may explain why they are well conserved. They appear to be ubiquitous among all angiosperms, including basal angiosperms, but are absent from non-angiosperms (Table S1, S2), suggesting that *MUG* was domesticated during early angiosperm evolution to perform what evolved to become a key function. Consistent with previous studies [17], [19], [24], it is clear from their phylogeny that the two *MUG* subfamilies diverged early in angiosperm evolution, prior to the monocot-eudicot split (Figure 1). However, phylogenetic and phenotypic differences between *MUGA* and *MUGB* suggest that they may have originated from more than one domestication event, similar to the *FHY3* family [30]. If all *MUG* genes did descend from a single domestication event, then while *MUGA* must have diversified prior to the monocot-eudicot split, *MUGB* must have acquired a PB1 exon and evolved rapidly prior to the monocot-eudicot split, yet not diversified until after the split. Alternately, if the *MUGB* subfamily descended from a different domestication event than *MUGA*, it may have acquired the PB1 domain either through transduplication prior to domestication [39], gene fusion during domestication [40], or exon shuffling subsequent to domestication. There may even have been more than two domestication events; in particular, Clade A3 has an unusual phylogenetic pattern, possibly reflecting a separate origin. Although multiple domestication events may help explain differences between the evolution and phenotypes of the two subfamilies, additional evidence, such as from phylogenetic studies of closely related MULEs or the functional characterization of additional *MUG* genes, will be required to resolve this question.

In summary, our results suggest the *MUG* family originated from TE genes adopting an adaptive function early in flowering plant evolution and are now conserved among angiosperms. Serious defects in *mug* mutants show that these genes make important contributions to fitness through roles in plant growth, flower development, and reproduction. The approach we used, of evaluating the fitness consequences of mutations to predicted DTEs, effectively couples *in silico* searches of genomic data with experimental validation. In the future, we expect that similar studies will enable a more complete characterization of TE-derived sequences that have been co-opted to provide fitness benefits.

## Materials and Methods

### *MUG* homologs

To confirm the protein sequences of the eight previously identified *A. thaliana MUG* genes [17], the sequences of *At-MUG1* and *At-MUG3* through *At-MUG7* were determined from gene models supported by publicly available full-length cDNAs at The Arabidopsis Information Resource (TAIR) database, release 10 [41]: *At-MUG1*, TAIR AT3G04605, GenBank AY074390; *At-MUG3*, AT1G06740, AK221278; *At-MUG4*, AT5G16505, AY059842; *At-MUG5*, AT3G06940, AF462806; *At-MUG6*, AT5G48965, AY136382; *At-MUG7*, AT3G05850, BT008628. *At-MUG2* (AT2G30640) and *At-MUG8* (AT5G34853) have no publicly available full-length cDNA sequence, so we predicted their structure from genomic DNA using FGENESH (dicot setting) [42]. The predicted sequence of *At-MUG2* was confirmed by RT-PCR (data not shown) and both *At-MUG2* and *At-MUG8* are consistent with available EST and mRNA-seq data [32], [41].

To identify additional *MUG* homologs, we performed comprehensive genomic searches in nine angiosperm genomes: *A. thaliana*, *Carica papaya*, *V. vinifera*, *M. truncatula*, *Mimulus guttatus*, *Sorghum bicolor*, *Z. mays*, *Oryza sativa* var. *japonica*, and *Brachypodium distachyon*. Because the identity between MUGA and MUGB sequences is low, a representative amino acid query was chosen for each subfamily: At-MUG1 for MUGA, At-MUG7 for MUGB.

In *A. thaliana*, *O. sativa*, and *S. bicolor*, we used BLASTP to search the protein databases of TAIR10 [41], the Rice Annotation Project (RAP-DB) [43], and Sbi 1.4 [44], respectively. To ensure that we found all *MUG* genes present in the datasets, we calibrated E-value thresholds to well below those needed to find all known *MUG* genes [17] as well as a few non-*MUG* sequences, and we validated the results using phylogenetic analyses. At each resulting locus, the highest-ranked annotated gene model was chosen. Conserved domains were identified using the NCBI Conserved Domain Database [45]. Gene models terminating in truncated domains were extended using FGENESH [42] where possible. Because TE-like genes (including DTEs) are commonly filtered from protein databases, we confirmed the results in *A. thaliana* using a TBLASTN [46] search of the TAIR10 Genes database, which includes loci annotated as “transposable element genes”. In *S. bicolor*, we confirmed the results using a TBLASTN [46] search of the unmasked genome assembly [44].

The remaining six genomes had limited gene models, so instead of searching protein databases, we searched whole genome assemblies: *C. papaya*, Hawaii Papaya Genome Project, 2007 release [47]; *V. vinifera*, Genoscope, March 2010, 12X assembly [48]; *M. truncatula*, Medicago Genome Sequencing Consortium, release Mt3.5 [49]; *M. guttatus*, Joint Genome Institute (JGI) assembly v1.0 and gene annotation v1.1 [50]; *Z. mays*, Maizesequence.org, release 4a.53 [51]; *B. distachyon*, JGI 8x assembly release v1.0 of strain Bd21 [50], [52]. We used TBLASTN [46] to identify potential *MUG* homologs and FGENESH [42] to predict corresponding gene models. Conserved domains were identified as above.

### Phylogenetic analyses

We conducted phylogenetic analyses on the amino acid sequences identified in the genome searches, including all putative MUG homologs as well as a sample of putative MURA sequences. Putative MURAs typically formed larger, low-identity clusters and the sequences often contained premature stop codons or frameshifts, even within conserved domains, which is characteristic of TEs. Clusters of putative MURA sequences with greater than 95% identity were represented by a single sequence. Alignments were generated using MUSCLE [53], curated with Gblocks [54], and phylogenetic analysis performed using PhyML (500 bootstraps) [55], [56]. All putative MURA sequences were non-monophyletic to the MUG tree. *Z. mays* MURA (GenBank AAA81535.1) was used as the outgroup. Synonymous substitution rates were calculated using PAML CODEML [57].

To determine the taxonomic distribution of *MUG* homologs, we conducted TBLASTN searches of NCBI dbEST libraries of each major seed plant subgroup not represented in the genome searches, using At-MUG2 and At-MUG7 queries as above (Table S2). We identified putative *MUG* ESTs by looking in each species for small clusters of top-ranked ESTs with low E-values and high identity, consistent with results of previously identified *MUG* homologs. We validated MUG and MURA assignments by phylogenetic analyses, using subsets of ESTs that could be aligned.

### Plant material

The mutants *mug1-1* (GK_514B01), *mug1-2* (GK_293B02), *mug2-3* (SALK_090878), *mug2-4* (SALK_055071), *mug7-1* (SALK_012814), *mug7-5* (GK_378C04), *mug8-1* (GK_244B09), and *mug8-2* (GK_155E09) were obtained from GABI-Kat (http://www.gabi-kat.de) [58] and SALK (http://www.arabidopsis.org/abrc) [59] T-DNA insertion populations. The positions of the insertion sites in double mutants used in the phenotypic analyses were confirmed by sequencing of the allele-specific PCR products (data not shown). Wild-type ecotype Col-0 seeds were originally obtained from Lehle Seeds (www.arabidopsis.com).

Seeds were sterilized using a 50% bleach solution for 5 min, then washed once in 95% ethanol and 3 times in sterile water. Sterilized seeds were sown on half-strength Murashige and Skoog (MS) [60] medium plates containing 0.8% agar (w/v) and 1% sucrose (w/v). Plated seeds were stratified in the dark for 3 days at 4°C and kept on plates for 2 weeks in a growth chamber (Conviron model E15) at 22°C under a 16 h light/8 h dark photoperiod, ∼100 µmol quanta/m^2^/s light intensity, 60% relative humidity. Seedlings were transplanted to soil with a composition of PRO-MIX (Premier Tech Horticulture, Quebec, Canada): vermiculite: perlite of 2∶1∶1 in 2 ½ inch square pots and returned to the growth chamber.

### Phenotypic analyses

We selected eight traits for phenotypic analyses previously shown to reflect plant fitness [61]–[65]. A detailed description of each trait is presented in Table S3. Statistical analysis was performed by two-sample *t*-tests using the wild-type control (α = 0.05).

Scanning electron microscopy was performed using a Hitachi S4700 Field Emission-STEM microscope. Wild-type and homozygous mutant inflorescences were fixed overnight in 2% glutaraldehyde, washed, and dehydrated using a series of graded ethanol solutions (30 to 100%). Dried samples were sputter coated with gold-platinum. Inflorescences were also photographed using an Olympus DP71 camera attached to an Olympus MVX10 stereomicroscope.

For the sucrose assay, Col-0 and mutant seeds were sown on MS medium plates with or without 100 mM sucrose. Seeds were stratified for 3 days in the dark at 4°C and transferred to a growth chamber with settings as above for 12 days with plates vertically-oriented, after which each plate was photographed using a Nikon D3100 camera and scored manually.

To measure total chlorophyll content, 200 mg of 17-day-old wild-type and mutant seedlings were extracted by shaking overnight in the dark in 1 ml of 80% acetone. Chlorophyll levels were measured using a 4050 Ultrospec II UV/Vis spectrophotometer (LKB Biochrom) and the total amount of chlorophyll was determined using MacKinney's coefficients [66] and the equation: chlorophyll a+b = 7.15×OD660 nm+18.71×OD647 nm.

## Supporting Information

Figure S1 Complete MUG sequences.
(PDF)

Figure S2 An alignment of MUG and MURA sequences. MUG sequences (A_M.G_10 and below) and MURA of MULEs (M_V.V_2 and above) are shown. Conserved domains are indicated by colored bars and active site residues by asterisks: MuDR (green), MULE (red), SWIM (orange). The CCHC residues of MuDR and CCCH residues of SWIM are conserved in MUG sequences; however, most have mutations to at least one of the DDE residues of the MULE domain.
(PDF)

Figure S3 *MUG* gene structure showing locations of T-DNA insertions and double mutant allelic combinations. (A) Graphical representation of *At-MUG1*, *At-MUG2*, *At-MUG7*, and *At-MUG8* gene transcripts with the position of the two T-DNA insertions for each gene. Bold horizontal lines represent transcripts, dipped lines represent introns, and large blocks represent regions encoding conserved protein domains. (B) Allelic combinations of the double mutants used in the phenotypic analysis.
(PDF)

Table S1 MUG sequences in basal angiosperms. Results of TBLASTN searches of At-MUG1 (MUGA) or At-MUG7 (MUGB) vs. EST consensus sequences from the Ancestral Angiosperm Genome Project (http://ancangio.uga.edu/content/est-assemblies). Counts are estimates based on similarity to At-MUG1 or At-MUG7, the presence or absence of a PB1 domain, the presence or absence of premature stop codons, and phylogenetic analysis.
(PDF)

Table S2 MUG sequences in dbEST. Summary of TBLASTN searches of At-MUG1 (MUGA) or At-MUG7 (MUGB) vs. EST sequences in NCBI dbEST.
(PDF)

Table S3 Detailed descriptions of fitness metrics.
(PDF)

Table S4 DDE motifs of MUG proteins. Only variant residues are shown.
(PDF)

Table S5 Locus IDs of *MUG* genes.
(PDF)

## References

[1] AGI (2000) Analysis of the genome sequence of the flowering plant Arabidopsis thaliana. Nature 408: 796–815. 11130711 10.1038/35048692

[2] Lander ES, Linton LM, Birren B, Nusbaum C, Zody MC, et al (2001) Initial sequencing and analysis of the human genome. Nature 409: 860–921. 11237011 10.1038/35057062

[3] Doolittle WF, Sapienza C (1980) Selfish genes, the phenotype paradigm and genome evolution. Nature 284: 601–603. 6245369 10.1038/284601a0

[4] Orgel LE, Crick FH (1980) Selfish DNA: the ultimate parasite. Nature 284: 604–607. 7366731 10.1038/284604a0

[5] Wicker T, Sabot F, Hua-Van A, Bennetzen JL, Capy P, et al (2007) A unified classification system for eukaryotic transposable elements. Nat Rev Genet 8: 973–982. 17984973 10.1038/nrg2165

[6] Volff J-N (2006) Turning junk into gold: domestication of transposable elements and the creation of new genes in eukaryotes. Bioessays 28: 913–922. 16937363 10.1002/bies.20452

[7] Feschotte C (2008) Transposable elements and the evolution of regulatory networks. Nat Rev Genet 397–405. 18368054 10.1038/nrg2337 PMC2596197

[8] Sinzelle L, Izsvak Z, Ivics Z (2009) Molecular domestication of transposable elements: From detrimental parasites to useful host genes. Cell Mol Life Sci 66: 1073–1093. 19132291 10.1007/s00018-009-8376-3 PMC11131479

[9] Volff JN (2009) Cellular genes derived from Gypsy/Ty3 retrotransposons in mammalian genomes. Ann N Y Acad Sci 1178: 233–243. 19845640 10.1111/j.1749-6632.2009.05005.x

[10] Hua-Van A, Le Rouzic A, Boutin TS, Filee J, Capy P (2011) The struggle for life of the genome's selfish architects. Biology direct 6: 19. 21414203 10.1186/1745-6150-6-19 PMC3072357

[11] Feschotte C, Pritham EJ (2007) DNA transposons and the evolution of eukaryotic genomes. Annu Rev Genet 41: 331–368. 18076328 10.1146/annurev.genet.40.110405.090448 PMC2167627

[12] Whitelam GC, Johnson E, Peng J, Carol P, Anderson ML, et al (1993) Phytochrome A null mutants of Arabidopsis display a wild-type phenotype in white light. Plant Cell 5: 757–768. 8364355 10.1105/tpc.5.7.757 PMC160314

[13] Hudson M, Ringli C, Boylan MT, Quail PH (1999) The FAR1 locus encodes a novel nuclear protein specific to phytochrome A signaling. Genes Dev 13: 2017–2027. 10444599 10.1101/gad.13.15.2017 PMC316922

[14] Hudson ME, Lisch DR, Quail PH (2003) The FHY3 and FAR1 genes encode transposase-related proteins involved in regulation of gene expression by the phytochrome A-signaling pathway. Plant J 34: 453–471. 12753585 10.1046/j.1365-313x.2003.01741.x

[15] Bundock P, Hooykaas P (2005) An Arabidopsis hAT-like transposase is essential for plant development. Nature 436: 282–284. 16015335 10.1038/nature03667

[16] Zdobnov E, Campillos M, Harrington E, Torrents D, Bork P (2005) Protein coding potential of retroviruses and other transposable elements in vertebrate genomes. Nucleic Acids Res 33: 946–954. 15716312 10.1093/nar/gki236 PMC549403

[17] Cowan R, Hoen D, Schoen D, Bureau T (2005) MUSTANG is a novel family of domesticated transposase genes found in diverse angiosperms. Mol Biol Evol 22: 2084–2089. 15987878 10.1093/molbev/msi202

[18] Muehlbauer GJ, Bhau BS, Syed NH, Heinen S, Cho S, et al (2006) A hAT superfamily transposase recruited by the cereal grass genome. Mol Genet Genomics 275: 553–563. 16468023 10.1007/s00438-006-0098-8

[19] Saccaro NL, Van Sluys M-A, de Mello Varani A, Rossi M (2007) MudrA-like sequences from rice and sugarcane cluster as two bona fide transposon clades and two domesticated transposases. Gene 392: 117–125. 17289300 10.1016/j.gene.2006.11.017

[20] Piriyapongsa J, Marino-Ramirez L, Jordan IK (2007) Origin and evolution of human microRNAs from transposable elements. Genetics 176: 1323–1337. 17435244 10.1534/genetics.107.072553 PMC1894593

[21] Benjak A, Forneck A, Casacuberta JM (2008) Genome-wide analysis of the “cut-and-paste” transposons of grapevine. PLoS ONE 3: e3107 doi:10.1371/journal.pone.0003107.. 18769592 10.1371/journal.pone.0003107 PMC2528002

[22] Le QH, Wright S, Yu Z, Bureau T (2000) Transposon diversity in Arabidopsis thaliana. Proc Natl Acad Sci U S A 97: 7376–7381. 10861007 10.1073/pnas.97.13.7376 PMC16553

[23] Yu Z, Wright SI, Bureau TE (2000) Mutator-like elements in Arabidopsis thaliana. Structure, diversity and evolution. Genetics 156: 2019–2031. 11102392 10.1093/genetics/156.4.2019 PMC1461377

[24] Rossi M, Araujo PG, de Jesus EM, Varani AM, Van Sluys MA (2004) Comparative analysis of Mutator -like transposases in sugarcane. Molecular genetics and genomics : MGG 272: 194–203. 15338280 10.1007/s00438-004-1036-2

[25] Boguski MS, Lowe TM, Tolstoshev CM (1993) dbEST–database for “expressed sequence tags”. Nat genet 4: 332–333. 8401577 10.1038/ng0893-332

[26] Babu MM, Iyer LM, Balaji S, Aravind L (2006) The natural history of the WRKY-GCM1 zinc fingers and the relationship between transcription factors and transposons. Nucleic Acids Res 34: 6505–6520. 17130173 10.1093/nar/gkl888 PMC1702500

[27] Hua-Van A, Capy P (2008) Analysis of the DDE Motif in the Mutator Superfamily. J Mol Evol 67: 670–681. 19018586 10.1007/s00239-008-9178-1

[28] Orr HA (2009) Fitness and its role in evolutionary genetics. Nature reviews Genetics 10: 531–539. 10.1038/nrg2603 PMC2753274 19546856

[29] Smith AM, Stitt M (2007) Coordination of carbon supply and plant growth. Plant, cell & environment 30: 1126–1149. 10.1111/j.1365-3040.2007.01708.x 17661751

[30] Lin R, Ding L, Casola C, Ripoll DR, Feschotte C, et al (2007) Transposase-derived transcription factors regulate light signaling in Arabidopsis. Science 318: 1302–1305. 18033885 10.1126/science.1146281 PMC2151751

[31] Simon SA, Meyers BC (2011) Small RNA-mediated epigenetic modifications in plants. Curr Opin Plant Biol 14: 148–155. 21159545 10.1016/j.pbi.2010.11.007

[32] Lister R, O'Malley RC, Tonti-Filippini J, Gregory BD, Berry CC, et al (2008) Highly integrated single-base resolution maps of the epigenome in Arabidopsis. Cell 133: 523–536. 18423832 10.1016/j.cell.2008.03.029 PMC2723732

[33] Zhang X, Yazaki J, Sundaresan A, Cokus S, Chan SW, et al (2006) Genome-wide high-resolution mapping and functional analysis of DNA methylation in arabidopsis. Cell 126: 1189–1201. 16949657 10.1016/j.cell.2006.08.003

[34] Gregory BD, O'Malley RC, Lister R, Urich MA, Tonti-Filippini J, et al (2008) A link between RNA metabolism and silencing affecting Arabidopsis development. Developmental cell 14: 854–866. 18486559 10.1016/j.devcel.2008.04.005

[35] de Araujo P, Rossi M, de Jesus E, Saccaro N, Kajihara D, et al (2005) Transcriptionally active transposable elements in recent hybrid sugarcane. Plant J 44: 707–717. 16297064 10.1111/j.1365-313X.2005.02579.x

[36] Jiao Y, Deng XW (2007) A genome-wide transcriptional activity survey of rice transposable element-related genes. Genome Biol 8: R28. 17326825 10.1186/gb-2007-8-2-r28 PMC1852403

[37] Ouyang X, Li J, Li G, Li B, Chen B, et al (2011) Genome-Wide Binding Site Analysis of FAR-RED ELONGATED HYPOCOTYL3 Reveals Its Novel Function in Arabidopsis Development. Plant Cell 23: 2514–2535. 21803941 10.1105/tpc.111.085126 PMC3226222

[38] Lin R, Teng Y, Park H-J, Ding L, Black C, et al (2008) Discrete and essential roles of the multiple domains of Arabidopsis FHY3 in mediating phytochrome A signal transduction. Plant Physiol 148: 981–992. 18715961 10.1104/pp.108.120436 PMC2556831

[39] Hoen DR, Park KC, Elrouby N, Yu Z, Mohabir N, et al (2006) Transposon-mediated expansion and diversification of a family of ULP-like genes. Mol Biol Evol 23: 1254–1268. 16581939 10.1093/molbev/msk015

[40] Cordaux R, Udit S, Batzer MA, Feschotte C (2006) Birth of a chimeric primate gene by capture of the transposase gene from a mobile element. P Natl Acad Sci U S A 103: 8101–8106. 10.1073/pnas.0601161103 PMC1472436 16672366

[41] Swarbreck D, Wilks C, Lamesch P, Berardini TZ, Garcia-Hernandez M, et al (2008) The Arabidopsis Information Resource (TAIR): gene structure and function annotation. Nucleic acids res 36: D1009–1014. 17986450 10.1093/nar/gkm965 PMC2238962

[42] Salamov AA, Solovyev VV (2000) Ab initio gene finding in Drosophila genomic DNA. Genome Res 10: 516–522. 10779491 10.1101/gr.10.4.516 PMC310882

[43] Tanaka T, Antonio BA, Kikuchi S, Matsumoto T, Nagamura Y, et al (2008) The Rice Annotation Project Database (RAP-DB): 2008 update. Nucleic Acids Res 36: D1028–1033. 18089549 10.1093/nar/gkm978 PMC2238920

[44] Paterson AH, Bowers JE, Bruggmann R, Dubchak I, Grimwood J, et al (2009) The Sorghum bicolor genome and the diversification of grasses. Nature 457: 551–556. 19189423 10.1038/nature07723

[45] Marchler-Bauer A, Lu S, Anderson JB, Chitsaz F, Derbyshire MK, et al (2011) CDD: a Conserved Domain Database for the functional annotation of proteins. Nucleic Acids Res 39: D225–229. 21109532 10.1093/nar/gkq1189 PMC3013737

[46] Altschul SF, Gish W, Miller W, Myers EW, Lipman DJ (1990) Basic local alignment search tool. Journal of molecular biology 215: 403–410. 2231712 10.1016/S0022-2836(05)80360-2

[47] Ming R, Hou S, Feng Y, Yu Q, Dionne-Laporte A, et al (2008) The draft genome of the transgenic tropical fruit tree papaya (Carica papaya Linnaeus). Nature 452: 991–996. 18432245 10.1038/nature06856 PMC2836516

[48] Jaillon O, Aury JM, Noel B, Policriti A, Clepet C, et al (2007) The grapevine genome sequence suggests ancestral hexaploidization in major angiosperm phyla. Nature 449: 463–467. 17721507 10.1038/nature06148

[49] Cannon SB, Sterck L, Rombauts S, Sato S, Cheung F, et al (2006) Legume genome evolution viewed through the Medicago truncatula and Lotus japonicus genomes. Proceedings of the National Academy of Sciences of the United States of America 103: 14959–14964. 17003129 10.1073/pnas.0603228103 PMC1578499

[50] Goodstein DM, Shu S, Howson R, Neupane R, Hayes RD, et al (2012) Phytozome: a comparative platform for green plant genomics. Nucleic acids research 40: D1178–1186. 22110026 10.1093/nar/gkr944 PMC3245001

[51] Schnable PS, Ware D, Fulton RS, Stein JC, Wei F, et al (2009) The B73 maize genome: complexity, diversity, and dynamics. Science 326: 1112–1115. 19965430 10.1126/science.1178534

[52] TIBI (2010) Genome sequencing and analysis of the model grass Brachypodium distachyon. Nature 463: 763–768. 20148030 10.1038/nature08747

[53] Edgar RC (2004) MUSCLE: multiple sequence alignment with high accuracy and high throughput. Nucleic acids research 32: 1792–1797. 15034147 10.1093/nar/gkh340 PMC390337

[54] Castresana J (2000) Selection of conserved blocks from multiple alignments for their use in phylogenetic analysis. Molecular biology and evolution 17: 540–552. 10742046 10.1093/oxfordjournals.molbev.a026334

[55] Dereeper A, Guignon V, Blanc G, Audic S, Buffet S, et al (2008) Phylogeny.fr: robust phylogenetic analysis for the non-specialist. Nucleic Acids Res 36: W465–469. 18424797 10.1093/nar/gkn180 PMC2447785

[56] Guindon S, Gascuel O (2003) A simple, fast, and accurate algorithm to estimate large phylogenies by maximum likelihood. Systematic biology 52: 696–704. 14530136 10.1080/10635150390235520

[57] Yang Z (2007) PAML 4: phylogenetic analysis by maximum likelihood. Mol Biol Evol 24: 1586–1591. 17483113 10.1093/molbev/msm088

[58] Rosso MG, Li Y, Strizhov N, Reiss B, Dekker K, et al (2003) An Arabidopsis thaliana T-DNA mutagenized population (GABI-Kat) for flanking sequence tag-based reverse genetics. Plant Mol Biol 53: 247–259. 14756321 10.1023/B:PLAN.0000009297.37235.4a

[59] Alonso JM, Stepanova AN, Leisse TJ, Kim CJ, Chen H, et al (2003) Genome-wide insertional mutagenesis of Arabidopsis thaliana. Science 301: 653–657. 12893945 10.1126/science.1086391

[60] Murashige T, Skoog F (1962) A Revised Medium for Rapid Growth and Bio Assays with Tobacco Tissue Cultures. Physiologia plantarum 15: 473–497.

[61] Alonso-Blanco C, Aarts M, Bentsink L, Keurentjes J, Reymond M, et al (2009) What has natural variation taught us about plant development, physiology, and adaptation? Plant Cell Online 21: 1877. 10.1105/tpc.109.068114 PMC2729614 19574434

[62] Shindo C, Bernasconi G, Hardtke CS (2008) Intraspecific competition reveals conditional fitness effects of single gene polymorphism at the Arabidopsis root growth regulator BRX. New Phytol 180: 71–80. 18627499 10.1111/j.1469-8137.2008.02553.x

[63] Donohue K (2002) Germination timing influences natural selection on life-history characters in Arabidopsis thaliana. Ecology 83: 1006–1016.

[64] Pigliucci M, Schlichting CD (1996) Reaction norms of Arabidopsis IV. Relationships between plasticity and fitness. Heredity 76 Pt 5:427–436. 8666543 10.1038/hdy.1996.65

[65] Shaw RG, Chang S-M (2006) Gene action of new mutations in Arabidopsis thaliana. Genetics 172: 1855–1865. 16361233 10.1534/genetics.105.050971 PMC1456307

[66] Mackinney G (1941) Absorption of light by chlorophyll solutions. J Biol Chem 140: 315–322.

