# Supplementary material for: A Gene Family Derived from Transposable Elements during Early Angiosperm Evolution Has Reproductive Fitness Benefits in Arabidopsis thaliana
Source: PLoS Genet. 2012 Sep 6;8(9):e1002931. doi: 10.1371/journal.pgen.1002931 (PMC3435246; doi:10.1371/journal.pgen.1002931)
Supplement: Figure S1 — Complete MUG sequences. (PDF) [file pgen.1002931.s001.pdf]

Figure S1.

>AtMUG1
MANRDLMLGQNRNIGIGQGQSLVLGHSHSLGLGQNHVDVELGQGHNMDLGLAHHDHDHHE
IDLGNPHDDHELDLGNSEDQDGEDHHHHHHHDYMNENEISVDQKPGHDDVDPDLVLSSQN
HEFSLSDNNHQLVVGENHELDDNLELAVDSSHELEIDQHGEMVMVSTPVQARALTADMTY
QLTVGQEFPDVKSCRRALRDMAIALHFEMQTIKSDKTRFTAKCSSDGCPWRVHAAKLPGV
PTFTIRTIHESHSCGGINHLGHQQASVQWVASSVEQRLRENPNCKPKEILEEIHRVHGIT
LSYKQAWRGKERIMATMRGSFEEGYRLLPQYCEQVKRTNPGSIASVYGSPADNCFQRLFI
SFQASIYGFLNACRPLLGLDRTYLKSKYLGTLLLATGFDGDGALFPLAFGIVDEENDENW
MWFLCELHNLLETNTENMPRLTILSDRQKGIVEGVEQNFPTAFHGFCMRHLSESFRKEFN
NTLLVNYLWEAAQALTVIEFEAKILEIEEISQDAAYWIRRIPPRLWATAYFEGQRFGHLT
ANIVESLNSWIAEASGLPIIQMMECIRRQLMTWFNERRETSMQWTSILVPTAERRVAEAL
ELARTYQVLRANEAEFEVISHEGNNIVDIRNRCCLCRGWQLYGLPCAHAVAALLSCRQNV
HRFTESCFTVATYRKTYSQTIHPIPDKSHWRELSEGDPNVNKAALDAIINPPKSLRPPGR
PRKRRVRAEDRGRVKRVVHCSRCNQTGHFRTTCAAPI
>AtMUG2
MTNNELIMSGNHDISFRQEQYMEARQNNELFTSHDYNFALEPLQETNLQIGVSREQNEVW
NHGQEHALALQNSGTHSEEIHEKQLVEGKDEVDSQENQNESYDHTFDLSFPDNDDSGTSE
NDKLALLVSQDLNDNLQLAVGHCMNRDPLPGLNISLSQQLELAPPILQCRSLQTAPEHNL
IVGMEFSDVLACRRALRDAAIALRFEMQTVKSDKTRFTAKCNSEGCPWRIHCAKLPGLPT
FTIRTIHGSHTCGGISHLGHHQASVQWVADAVTERLKVNPHCKPKEILEQIHQVHGITLT
YKQAWRGKERIMAAVRGSYEEDYRLLPRYCDQIRRTNPGSVAVVHGSPVDGSFQQFFISF
QASICGFLNACRPLIGLDRTVLKSKYVGTLLLATGFDGEGAVFPLAFAIISEENDSSWQW
FLSELRQLLEVNSENMPKLTILSSRDQSIVDGVDTNFPTAFHGLCVHCLTESVRTQFNNS
ILVNLVWEAAKCLTDFEFEGKMGEIAQISPEAASWIRNIQHSQWATYCFEGTRFGHLTAN
VSESLNSWVQDASGLPIIQMLESIRRQLMTLFNERRETSMQWSGMLVPSAERHVLEAIEE
CRLYPVHKANEAQFEVMTSEGKWIVDIRCRTCYCRGWELYGLPCSHAVAALLACRQNVYR
FTESYFTVANYRRTYAETIHPVPDKTEWKTTEPAGESGEDGEEIVIRPPRDLRQPPRPKK
RRSQGEDRGRQKRVVRCSRCNQAGHFRTTCTAPI
>AtMUG3
MANDELVKSENRDISFRQDHFTGSRHNYELFVGHDYNSAFEQLLDINLQIGASCNGNELW
NNLQDHRGIDETKEVACLDVNQNDGYRSFPDNDDSEIDKSTVIEATGAIHKVESQDTDDK
LELEMSQSTEFSQKSVMPSPSSQCWSMSGAGTDHEMVVGMEFSDAYACRRAIKNAAISLR
FEMRTIKSDKTRFTAKCNSKGCPWRIHCAKVSNAPTFTIRTIHGSHTCGGISHLGHQQAS
VQWVADVVAEKLKENPHFKPKEILEEIYRVHGISLSYKQAWRGKERIMATLRGSTLRGSF
EEEYRLLPQYCDEIRRSNPGSVAVVHVNPIDGCFQHLFISFQASISGFLNACRPLIALDS
TVLKSKYPGTLLLATGFDGDGAVFPLAFAIVNEENDDNWHRFLSELRKILDENMPKLTIL
SSGERPVVDGVEANFPAAFHGFCLHYLTERFQREFQSSVLVDLFWEAAHCLTVLEFKSKI
NKIEQISPEASLWIQNKSPARWASSYFEGTRFGQLTANVITESLSNWVEDTSGLPIIQTM
ECIHRHLINMLKERRETSLHWSNVLVPSAEKQMLAAIEQSRAHRVYRANEAEFEVMTCEG
NVVVNIENCSCLCGRWQVYGLPCSHAVGALLSCEEDVYRYTESCFTVENYRRAYAETLEP
ISDKVQWKENDSERDSENVIKTPKAMKGAPRKRRVRAEDRDRVRRVVHCGRCNQTGHFRT
TCTAPM
>AtMUG4
MADGALITLDSDDILATEHVLAVGKEFPDVETCRRTLKDLAIALHFDLRIVKSDRSRFIA
KCSKEGCPWRIHAAKCPGVQTFTVRTLNSEHTCEGVRDLHHQQASVGWVARSVEARIRDN
PQYKPKEILQDIRDEHGVAVSYMQAWRGKERSMAALHGTYEEGYRFLPAYCEQIKLVNPG
SFASVSALGPENCFQRLFIAYRACISGFFSSCRPLLELDRAHLKGKYLGAILCAAAVDAD
DGLFPLAIAIVDNESDENWSWFLSELRKLLGMNTDSMPKLTILSERQSAVVEAVETHFPT
AFHGFCLRYVSENFRDTFKNTKLVNIFWSAVYALTPAEFETKSNEMIEISQDVVQWFELY
LPHLWAVAYFQGVRYGHFGLGITEVLYNWALECHELPIIQMMEHIRHQISSWFDNRRELS
MGWNSILVPSAERRITEAVADARCYQVLRANEVEFEIVSTERTNIVDIRTRDCSCRRWQI
YGLPCAHAAAALISCGRNVHLFAEPCFTVSSYQQTYSQMIGEIPDRSLWKDEGEEAGGGV

ESLRIRPPKTRRPPGRPKKKVVRVENLKRPKRIVQCGRCHLLGHSQKKCTQPI
>AtMUG5
MTTNKVIAICQSGGEFVTIKDGSLSYSGGDAFAIDIDQNTSMTDFKSELAENFGFGLEAM
TLKYFLPGNKKTLITISKDKDFIRMVNFSSDAGTVEIFVIPEEAAAKNLSVMPASRSSRT
TASEGVVPAICAVGDGVEEDMANDFQIEMAMPDDSPLPCNFVLVDQRHHTAIQQWENVIT
GVDQRFNTFLEFRDALHKYSVAHGFAYKYKKNDSHRVSVKCKAQGCPWRITASRLSTTQL
ICIKKMNPRHTCERAVIKPGYRATRGWVRTILKEKLKAFPDYKPKDIAEDIKKEYGIQLN
YSQAWRAKEIAREQLQGSYKEAYSQLPLICEKIKETNPGSIATFMTKEDSSFHRLFISFY
ASISGFKQGSRPLLFLDNAILNSKYQGVMLVATASDAEDGIFPVAFAIVDSETEENWLWF
LEQLKTALSESRIITFVADFQNGLKNAIAQVFEKDAHHAYCLGQLAEKLNVDLKGQFSHE
ARRYMLNDFYSVAYATTPVGYYLALENIKSISPDAYNWVIESEPHHWANALFQGERYNKM
NSNFGKDFYSWVSEAHEFPITQMIDEFRAKMMQSIYTRQVKSREWVTTLTPSNEEKLQKE
IELAQLLQVSSPEGSLFQVNGGESSVSIVDINQCDCDCKTWRLTGLPCSHAIAVIGCIEK
SPYEYCSTYLTVESHRLMYAESIQPVPNMDRMMLDDPPEGLVCVTPPPTRRTPGRPKIKK
VEPLDMMKRQLQCSKCKGLGHNKKTCKAT
>AtMUG6
MATKKVIAICQSGGEFVTNKDGSLAYSGGDAYAIDIDQDTCMSDFKSELAENFGFSWENM
TLKYFLPGNKKTLITISKDKDFQRMVSFSADAPNVEIFVLPEEAEARNVSNMPASRSSRT
TASEAVVPVVSAGDIVVGDDMIANDFQIEMGIMPEESSPLPCNFVLTDEKQHIKAAQQWE
NAITGVDQRFNSFTEFRDALHKYSIAHGFTYKYKKNDSHRVSVKCKAQGCPWRITASRLS
TTQLICIKKMNTRHTCERAVVKAGYRASRGWVGSIIKEKLKAFPDYKPKDIAEDIKREYG
IQLNYSQAWRAKEIAREQLQGSYKKAYSQLPSFCKKIRETNPGSIAIFMTKEDSSFHRLF
ISFYASISGFRQGCRPLLFLDTADLNSKYQGVMLVATAPDAEDGIFPVAFAVVDAETEDN
WVWFLEHLKKLALADPRTITFVADFQNGLKTALPLVFEKQHHHAYCLRHLAEKLNMDLQAQ
FSHEARRFILNDFYAAAYATQPDAYYRSLENIKSISPDAYTWVIESEPLHWANALFEGER
YNHMNSIFGLDFYSWVSEAHELPITQMIDELRAKLMQSIYTHQVQSREWIVSTLTPTNEE
KLQKEIELARSLQVSAPHNSLFEVHGETINLVDINQCDCDCKVWRLTGLPCSHAVAVVEC
IGKSPYEYCSRYFTSESYRLTYAESINPVPNTTMTMMILEEPPVEGVVSVTPPPTRLTPP
GRPKSKQVEPLDMFKRQLQCSNCKGLGHNKKTCKAVS
>AtMUG7
MSGKRVIAICMSGGEFQTEKGGSLSYKGGDAHAIDVDEQMKFIDFISEIGEMFNCDVRTV
SLKYFLPDNKKTLISISNDKDLKRMIKFHENSNTADVYLLPEEATPDISNMPASRSSRTT
LSEAIPPVPMDDMIDDTMGPEELPISISVSAPPAVTMEEVMNRAEDAQIIINPSELMSSI
VEVPNPKGDILTKARTQQWQNTITGVGQRFKNVGEFREALRKYAIANQFGFRYKKNDSHR
VTVKCKAEGCPWRIHASRLSTTQLICIKKMNPTHTCEGAGGINGLQTSRSWVASIIKEKL
KVFPNYKPKDIVSDIKEEYGIQLNYFQAWRGKEIAREQLQGSYKDGYKQLPLFCEKIMET
NPGSLATFTTKEDSSFHRVFVSFHASVHGFLEACRPLVFLDSMQLKSKYQGTLLAATSVD
GDDEVFPLAFAVVDAETDDNWEWFLLQLRSLLSTPCYITFVADRQKNLQESIPKVFEKSF
HAYCLRYLTDELIKDLKGPFSHEIKRLIVDDFYSAAYAPRADSFERHVENIKGLSPEAYD
WIVQKSQPDHWANAYFRGARYNHMTSHSGEPFFSWASDANDLPITQMVDVIRGKIMGLIH
VRRISANEANGNLTPSMEVKLEKESLRAQTVHVAPSADNNLFQVRGETYELVNMAECDCS
CKGWQLTGLPCHHAVAVINYYGRNPYDYCSKYFTVAYYRSTYAQSINPVPLLEGEMCRES
SGGSAVTVTPPPTRRPPGRPPKKKTPAEEVMKRQLQCSRCKGLGHNKSTCKDYLLEC
>AtMUG8
MGKGKLILICQSGGKFVTDDDGTMTYTGGEAEAIDINHETTFDDFKLKLAKLLNLAYSSL
SLKYFLPGNRRTLITMKQEKDMKRMYDFHLSSVTAEVFITGQYGFQSEAVLSPGTRSYNI
AIGTETTPVAYGNVANVPIQVITGTPEENNLADINLSSRKVAPRVTSESSGLIDIPVTIS
TIPVGPAKSTSKKFKTKGKNSLVSNITNLTPKSLKQTFIGSNPGSKSSPPTSLSVVCGVT
TSSPRSVSKRRRVMEEPIILLQDENVTDTRRRSLRNRGEIRKPVIETDDDDVDADEVDSD
EDDSDDVDADNVEDDDDKDYVQDIETYYPETEDLDYEREINYSISEANDGSVESLVASWK
RCITGVGQGFESVVEFRDALQKYAVACRFGYRLRKNESNRACGVCLVGGCPWKIYASWVP
SESVFRIKKFNRRHTCGGESWKSAHPKKNWVVGIIKERLQENPNQKTKNIADSFFQDFGI
ELSYCTIRRGIDEAKGGLHTSFKEAYKYLPHFVNKVVEANPGSMVNLVVGEDRRFQRLFL
SFQSCIHGFQTGCRPLLFLDAIPFKSRYHEILLTASALDGDDCVLPVALALVDVETDETW

RWFLEQLKVALSSLRPLTFVSDREKGLVTSVLEIFENAHHGYSIHYLMEDFMRSLRGPFL
GDGKPSLTYYLLAAARADRLDGFKVYTEQIRRVSPRAYDWVMQIESKHWACALFEGEPYS
HITSDVAEIYSKWIEEIQETSIVQKLVAFVNKIVELVNSSQEKSKPWFSQLVPSKEESLV
EECKKAGSLKVFFCSDTLFEVHDGSVQLVDISNQTCSCFGWKPTGLPCQHAIAVLNTKGR
NLYDYCSSFFTVDSYRLTYSVALGAVAIDLALVENEGSGKEEDEQVLPPLFSRVQGVEKR
IKDRKRGRSVCCTKCGGVGHNKATCKDD
>Os1
MANHDLVLGHGEDPELALGQNHHDFGQDHGLGLGHSHELGLGHAHEHDLVLGQSHEHEHE
HDHDLGLGNHHDSQLVLAHDHHGHTSELALGHGHDEDPHSLDGQDHDGLAMTENHVLTLT
DAHQLDVDQNMDQLSLEQAHELALQPAHDFSHGPLAVAPVVQSRKMVVSSEFQLVVGQEF
PDVMSCRRAIRNTAIACHFEIQTVKSDKTRFTAKCSADGCPWRIHAAKLPGVPTFSIRTI
HDNHSCVGINHLGHQQASVQWVANTVEERLRENPHCKPKEILEEIHKSHGITLSYKQAWR
GKERIMAAVRGSFEEGYRLLPEYCRQVERTNPGSIARVYGNPDDNCFRRLFISFHASIYG
FVNACRPLIGLDRTILKNKYLGTLFLATGFDGDGALFPLAFGVVDEESDENWIWFLSELH
ELLEKNTENMPRLTILSDRRKGIIDGVDFNFPTAFHGHCMRSLSETFRKEFNNSVLVNLL
WEAANALTVIEFETKLLEIEDTSPEAVCWIRRLPPRLWATAYFEGTRYGHLTANITESLN
SWILDASGLPIIQMMECIRRQLMTWFNERREASMQWTTILVPAAERRVQEAIERARGYQV
ARANEAEFEVISPHEGTNIVDIRNRCCLCRGWQLYGVPCAHGVAALLSCRQNVHRYTESC
FTVATYRKTYSQTIHPIPDKTLWNETSDQGQEEENKVDVIINPPKSLRPPGRPRKKRIRA
EDRGRIKRVVHCSRCNQTGHFRTTCAAPI
>Os2
MGSKQIVAVLQVGGEFKTDDDGQMSYSGGEAHAMHVKSDWTFKTFKHEISSTLNNLKLDS
YVFKYFLPRNNKTLISISNDKDLKRMVEFHAESETTYIYVMKKADNRVKTSAVVPAASAD
HAVAATTPDGSKRQKICASWENAITGAGQVFEGPKEFRDALHKYAIAHRFHYRFVKNDSS
RVTVECTAEGCPWRIHASKSPAKKDFMIKKVFGSHTCESESVKSHRLASQKWVASVIKEK
LRDSPNYRPRDIANDLQREYGLSLNYSQAWRGKSIAQKELYSSHEEACNQLPWFCQRIVE
TNPGSAATVEALEDSKFRFFVAFHASIQGFVHGCRPLLFLDVISVKPNKHWKLLAATSVD
GEGDMFPVALSVVDDESQENWHWFLEQLKASLPVSGELTFISNGKCGLLDEVSLIFPDSY
HGYHVNYFIEEFKAQLDDSWSEELKDTMVEHVKKAMYSCKVDEFNQCIELIKVESDKLAE
WLLETKPEKWSDAFFKGSRLGQYTCNVPETILQWVPSRYELSVVQLVDTIRCNLMEMMYT
RREYSNSWTEPLTPSTNQKIQEEMGKALTHSVVCSTGNDGNNNVFEVCDGAVNVVNIDTW
DCTCRKWHVSGIPCSHAIAVFERTDHNPLDFCAKYFTTECYRLTYAMSINPIPDIVVAAP
STDPSQGEALHQSPILTRRQVGRPKEKPADPRIAIKRAVRCSRCKGYGHNKATCKVPIAA
>Os3
MGKGEIVAVLQVGGEFSTDADGLMSYSGGEAHAMLVKSDWTFSAFKHEISSTLNNIRVDQ
FVFKYFLPKNKKTLISISNDKDLHRMVEFHAESETTYIYVMKKKVDNRASIVVADSGTPV
DTTAVVPTTQDGSKRQKICATWKNVITGVGQVFDGPKDFRDALHKYAIAHKFHYRFIKND
SSRVTAECTGEDCPWRIHASKSPAKKQFMIKKISESHTCESETVKSHRLASQRWVASVIK
EKLRDSPNYRPRDIASDLQREYGLCLNYSQAWRGRSIAQKELHSTHDEVTSQLPWFCGRI
MQTNPQSVATVVPMEDSKFRLFVAFHASLHGFEHGCRPLLFLDVISAKPNKQWKLLTATS
VDSEGDVFPVAFAVVDDESSEHWHWFLEQLKSSVSASRAITFISNGENGLWDEVPLVFPD
SHHGYCVDYLIEEFKTQLDDAWTEEARDVMVEHLKKAIYACTVDEFNQYIELIKGESDKL
AEWLLEIKPERWSDAFFKGSRHGQYSCNFPSTIVEWIPTRYELSIVQLVDMIRCKLMEMM
YTRRESSNAWAELLTPAANQKLQEEVSKAHTLNVIPSESNENGNVFKVCDDSVNVVNIDA
WECTCRKWHISGLPCMHAIAVIERIGHCPYDYCVKYFRTECYRLTYSMSINPIPDVMLPP
AILSDQSQSPVTHATPIRTRRRVGRPKEKPADPRIAIKRAVRCSRCQGYGHNKATCKVPL
ST
>Os5
MEDDQILVEPDLAAELEQSLVDPTHELEQNLVEQGLVIGQEFVDVHACRRAVKDMAIAMH
FELRVVKSDRSRFIAKCAREGCPWRVHVAKCHGVPTFTVRTLHGEHTCDGVRDLHHHQAT
VGWVARSVEATLRDNPQYKPKEILQDIREQHGVAVSYMQAWRGKERSMAAVHGTLEDGYR
FLPAYCEQIVQTNPGSVAIYKGTGPDNSFQRLFVSFHASIHGFLNACRPLLEIDKADLKG
KYLGTLLCASAVDAENMMFPLAFGIVDAESDENWMWFFSELRKMLGVNTDKMPVLTILSE
RQSQVVEAVEVNFPTAFHGFCLRYVSENFRDEFKNPKLLNIFWSAVYALTAAEFDSKVND

MVQVQDVMPWFQRFPPNLWAVSYFEGIRYGHFNLGITEILYNWAMECHEFPIVQTVEHIK
HQLTCWFVERQNLALSYNSILVPSAEKLISEAIADSGCYQVLRANKVEFEIVSSERTNIV
DTQA
>Os6
MAEGSLVVAICQYGGEFTSGPNGNLIYKGGEAHAVDVTREMSLDNFKDEVSKVFHVEVSD
VSLKYFLPNNNRTLITISCDRDLQRMVDFTASSAQVDVFLISRVENRSITQTGASTAKPG
SNARGDKRKTPTSKNKASKNKKKTPSATGTAVQANANNVKQPRQVVTENDDNRVFPLEFG
SDIAFANTAGAGSTAPDILNQQKLALVDNTARESVGLFDDSVNPYVGSEITTEPTQGLNN
PIVFWDDIIKGVGQEFDNVKDFRAQLCKYAIGKGFVYRFIKNETTRVTVKCVGEGCTWRL
HASESSRNKKFVIKKMTDEHTCGGGSGEGQRRATRQWLTTVIKEKLHENPLFKPKDLVKE
IYEEYGVMLTYSQVWRGREVAQKELYHAIRETYSHLPWYCERLLETNPGSIALLSPMVDT
KFRRFFVAFHASLHGFTNGCRPLIFLDKVPLKATNEYKLLVAAGVDADDGVFPVAFNVVE
DENYESWVWFLMQLRYALQNHNYPYNAMTFLSSGQKGLDAAVPQVFEESHHAFCLHHIME
EFKGELRKGPWSQQIRDGMVEDFTRAAQACSIEDFNASIESIRNISTEAADWIIASKPEH
WSDAIFRGCRYDHFSSNIVDAFNNWIPTKKEGSIVLMIDSLRMKIMEVIEARRESCKSWS
GPLTPSMEFKVQDEMSKAGKLTVLCSSETVFEVRGSAIYVVNLANWECTCRRWQLSGLPC
MHAVAVFNRVGRSFYDYCSKFFRIESYHLAYSGAIFPIPDMDTVDFSAGANLIPPPKPRT
SDKPRRKRFNPNKIPTVVRLCSRCKQAGHNKATCEAIL
>Mt1
MDQKPEHDGNDELSFPEQNHELVLSENNDLTVSESQGFNESMDLAVVQNPEMSIESANDM
VVYQSQYMLSSTPHVVQARTVDVIPTYELSVGQEFPNVKSCRRALRDTAIVLHFEMQTIK
SDKTRFTAKCASEGCPRRIHAAKLPGVPTFTIRTIRESHTCGGISHLGHQQASVQWVASS
VEQRLNENPNCKPKEILEEIHRVHGITLSYKQAWRGKEHIMAAMRGSFEEGYRLLPQYCA
HVKRTNPGSIASVYGNPSDNCFQRLFISFQASIYGLLNACRPLLGLDRIYLKSKYLGTLL
LATGFDGDGALFPLAFGVVDEENDDNWMWFLSKLHNLLEINTENMPRLTILSDRQQGIVD
GVEANFPTAFHGFCMRHLSDNFRKEFNNTMLVNLLWEAANALTIIEFEGKVMEIEEISQD
AAYWIRRIPPRLWATAYFEGQRFGQTTANIVEDLNSWILEASGLPIIQMLECIRRQLMTW
LYERRETSMQWASVLVPSAERRVTEAIEHARTYQVLRANDAEFEVISHEGTNIIDIRNRC
CLCRGWQLYGLPCAHAVAALLSCRQNFHRFTESCFTVATYRKTYSETIHPIPDKSLWKEL
SEGDASVSQALEVVIYPPKSLRPPGRPRKKRVCAEDRGRVKRVVHCSRCNQTGHFRTTCA
API
>Mt2
MDNHSLVLDDTSVAIAEQPLVIGQEFPDVETCRRTLKDIAIAMHFDLRIVKSDRSRFIAK
CSKEGCPWRVHVAKCPGVPTFTIRTLQADHTCEGVRNLHHQQASVGWVARSVESRIRDNP
QVKPREILQDIRDQHGVAVSYMQAWRGKERSMAALHGTFEEGYRLLPAYCEQIRKTNPGS
IASVGATGQENCFQRLFISYRASIYGFINACRPLLELDRAHLKGKYLGSILCAAAVDADD
ALFPLAIAVVDTESDENWMWFMSELRKLLGVNTDNMPRLTILSERQRGMVEAVETHFPSA
SHGFCLRYVSENFRDTFKNTKLVNIFWNAVYALTAAEFESKITEMIEVSQDVISWFQHFP
PFLWAVAYFDGVRYGHFTLGVTELLYNWALECHELPVVQMMEYIRQQMTSWFNDRREVGM
EWTSILVPSAEKRISEAIADAHCYQVLRANEVEFEIVSTERTNIVDIRSRECSCRRWQLY
GLPCAHAAAALISCGHNAHMFAEPCFTVQSYRMAYSQMINPIPDKSQWREHGEGAEGGGG
ARVDIVIHPPKIRRPPGRPKKKVLRVENFKRPKRVVQCGRCHMLGHSQKKCTMPI
>Mt3
MAATATKKVIAICQSRGEFVTNIDGSMSYNGGDAYAIDIDQETSLSDFKSEIAETFNCNV
STMNIKYFLPGNKKTLITVSKDKDLQRMVSFLGDASTVDVFVINEEVVARNTSNMPASRS
SRTTVSEAVVPVVIPINVAAIDAEQCIDQVEVDVANEAPAQSLCSGANDDKRQRAAQQWE
NTITGVDQRFNSFSEFREALHKYSIAHGFAYRYKKNDSHRVTVKCKSQGCPWRIYASKLS
TTQLICIKKMTRDHTCEGSAVKAGYRATRGWVGNIIKEKLKASPNYRPKDIADDIKREYG
IQLNYSQAWRAKEIAREQLQGSYKEAYTQLPFFCEKIKETNPGSFATFTTKEDSSFHRLF
VSFHASITGFRQACRPLIFLDSILLNSKYQGELLAATSVDGNDGIFPVAFAVVDAETEDN
WHWFLQELKSALSTSEQITFVADFQNGLKKSLSEIFENCYHGYCLRHLADKLNKDLKGQL
SHEARRFMVNDFYAAAYASKLEIFERSIENIKGISPEAYNWVIQSEPEHWSNAFFNGARY
NLMTSNFGQQFYSWVSEANELPITQMIDVLRGNMMETISTRREKSNQWITKLTPSKEEII
QKETSDARSLQVLLSQGTTFEVCGQSVEIVDIDNWDCSCKGWKLTGLPCCHAIAVFECVG

RDLYDYCSRYLTVDNYRLTYTEPIHALPDIDKPVQVESAMEVVTVTPPPTKRPPGRPKSK
QVESIDLIKRQLQCGKCKGLGHNRKTCKLS
>Mt6
MDKRWGDWTLQIILVIWEKRGNADGCFSEESFKLFCVYDRKDDAVTEFPDVKAFRNAIKE
AAIAQHFELRIIKSDLIRYFAKCASEGCPWRIRAVKLPNASTFTIRSLEGTHTCGRNALN
GHHQASVDWIVSFIEERLRDNINYKPKDILHDIHKQYGITIPYKQAWRAKERGLAAIYGS
SEEGFYLLPSFCEEIKKTNPGSVAEVFTTGADSRFQRLFISFYASIHGFVNGCLPIVALG
GIQLKSKYLSTFLSATSFDADGGLFPLAFAVVDVENDESWTWFLSELHNALEVNTECMPQ
IIFLSDGQKGIVDAIRRKFPRSSHAFCMRHLSENIGKEFKNSRLIHLLWSAAYATTINAF
REKMAEIEEVSPNASMWLQHFHPSQWALVYFEGTRYGHLSSNIEEFNKWILEAQELPIIQ
VIERIQSKLKTEFDDRRLKSSSWCSVLTPSSERRMVEAINRASTYQVLKSDEVEFEVISA
DRSDIVNIGSHSCSCRDWQLYGIPCSHAVAALISSRKDVYAYTAKCFTVASYRDTYAEVL
HPVPGKLEWRTDESALDNDIAVVRPPKFRRPPGRPEKKRICVEDHNRDKHTVHCSRCNQT
GHYKTTCKAEMISSIEQF
>Cp1
MADPTLIVPDSSQTLIGQTLVIGQEFPDVETCRRTLKDIAIALHFDLRIVKSDRSRFIAK
CSKEGCPWRVHVAKCPGVPTFSIRTLHGEHTCEGVRNLHHQQASVGWVARSVEARIRDNP
QYKPKEILQDIRDQHGVAVSYMQAWRGKERSMAALHGTFEEGYRVLPAYCEQIRKSNPGS
IASVFAAGPENCFQRLFISYRASIYGFINACRPLLELDRAHLKGKYLGTLLCAAAVDADD
ALFPLAIAIVDVESEENWMWFMSELRKLLGVNTDSMPRLTILSERQRGIVEAVETHFPTA
FHGFCLRYVSENFRDTFKNTKLVNIFWNAVYALTAAEFEAKISEMVDISQDVLPWFQQFP
PQLWAVAYFEGVRYGHFALGVTELLYNWALECHELPVVQMMEHIRHQLAAWFDDRRDMGM
RLNSILVPSAEKRISEAIADARCYQVLRANEVEFEIVSTERTNIVDIRSRVCSCRRWQLY
GVPCAHAAAALISCGQNAHLFAEHCFTVASYRETYSQMINPIPDKSLWKEQGEGTEGGGA
NLDITIHPPKTRRPPGRPKKKVLRVENLKRPKRIVQCGRCHLLGHSQKKCTMPI
>Cp3
MGTHGNPNLGWEHANGDGLELGYTKENHIAPQNPYIHQVKDDGEYLDRVDNPAGVNNLNG
DIQAYDNQLALSHHLALSENHEMTNVGFGNQDVAENVDVNMEENQERSNVVTTESVEQAE
LVLDPPVLQIRSLAQMENHELTIGQEFPDAKSCRRAVRNAAIALRFEVQTFKSDKTRFTA
KCATEGCPWRIHAAKLPGLPTFSIRTIHDHHTCVGIAHLGHQQASVQWVADTLAEHIKQN
PQCKPKEILEEIHRVHGITLSYKQAWRGKERIMAAIRGSFEEDYRLLPQYCDQVQKANPG
SIALVYSNPNDGSFQHLFISFQASIYGFLNACRPLIGLDKTCLKSKDLGSILLATGFDGE
GAVFPLAFAVVDEENDDNWTWFLFELHKLLEINTDNMPKLTILSNRKRCIVDGVEANFPT
AFHGFCLHYLAESFHKEFNNSILLPLFWEAAAALTVIEFETKILEIQHISPSAALWIQNI
PYTLWTTSHFEGK
>Cp4
MEHHTFVVGQEFPDVKAFRNAIKEAAIAQHFELRIIKSDLIRYFAKCATEGCPWRIRAVK
LPNAPTFTIRSLEGTHTCGKNAQNGHHQASVDWIVNFIEERLRDDINYKPKDILQDIHKH
YGITIPYKQAWRAKERGLAAIYGSSEEGYCLLPSYCEHIKKTNPGSIAEVFTTGADNRFQ
RLFVSFYGSIYGFLHGCLPIVCLGGIHLNSKYLGTLLSATSFDADGGMFPLAFGVVDVEN
DDSWMWFLSELHRALEINAENMPQLTFLSDVQKGITDAVRRKFPTSSHAICMRHLSESIG
KEFKNSRLVNLLWKAAYATTTIGFKERMAEIYEVSSEAAKWIQQFPPPRWALAYFEGTRY
GHLSSNIEEFNRWILEARELPIIQVIERTHSKLIDEFEERRMKSASWFSVLAPSAENRMV
EAISRASTYQVLRSDEVEFEVLSADRSDIVNIGMQSCSCRDWQLYGIPYYQEIFVYHRAD
VYCPDYVTKELPSSLEWRLV
>Cp5
MAAKKIIAICQSGGDFVTNRDGSLSYTGGDAYAIDIDQHTQLDAFKAELAEMFACSIDTI
SIKYFLPGNKKTLITISKDKDFNRMVNFSGDAGTVEIFVMSEEAAARNVSNMPASRSSRT
TVSEAAVLAVAPVDAVIDMPNVIDRVDMDIHNETPLACMSPDMIEDRHHKAAQQWENTIT
GVDQRFNSFSEFREALHKYSIAHGFAYRYKKNDSHRVTVKCKSQGCPWRIYASRLSTTQL
ICIKRMSTRHTCEGVTVKAGYRATRGWVGSIIKEKLKVSPNYKPKDIADDIKREYGIQLN
YSQAWRAKEIAREQLQGSYKEAYSQLPFFCEKIKETNPGSITTFTTKEDSSFHRLFVSFH
ASISGFQQGCRPLLFLDSTALNSKYQGVLLVATAPDAEDGIFPVAFAVVDAETEDNWHWF
LVELKSAVSESRRITFVADFQNGLKRSLAEVFERCYHSYCLRHLAEKLNRDLKGQFSHEA

RRFMINDFYAAAYAPKLEGFQNSVENIKGISPEAYNWVVQSEPEHWANALFEGERYNHMT
SNFGQVFYSWVSEAHELPITQMIDSLRGKMMEAIYMRRVESNQWETTLTPSNEEKLQKET
ALARSLQVLLSHGSTFEVRGDSVDIVDVDHCECSCRSWRLSGLPCCHAIAVFECIGKNPY
EYCSRYLTTESYRLSYAESIYPVPNVDRSLLDESSQMVVTVTPPPTKRPPGRPKMKQAES
MDIIKRQLQCSKCKGLGHNKKTCKGS
>Cp6
MAGKKIIAICMSGGEFETEKDGSLLYKGGDAHAIDIDDQIKFNDFKMEVAEMFNYSNDTM
SMKYFLPGNKKTLITISSDKDLKRMIKFHGNCDTADIYIIMEETVALDVSNMPASRSSRT
TLSEAMPQISRPLSNEDDVMDDSTQPEISLIVPLDVVEETNHIVADVDDAHLNIPAEISP
MLPLASSNDEKHAKGAQQWQNTITGVGQRFSSVHEFRESLRKYAIAHQFAFRYKKNDSHR
VTVKCKAEGCPWRIHASRLSTTQLICIKKMNPTHTCEGAVVTTGHQATRSWVASIIKEKL
KVFPNYKPKDIVNDIKQEYGIQLNYFQAWRGKEIAKEQLQGSFKDAYNQLPLFCEKIMEL
NPGSLATFTTKEDSSFHRLFVAFHASLYGFLQGCRPLLFLDSIALKSKYQGLVLLMGMME
YFPVAFAIVDTDTDDNWALLKSALSTSCPLTFVADRQKGLRESIAEIFKGSFHAYCLRYL
TEELIRDLKGQFSHEVKRLMVEDLYGAAYAPSSEGFQRCVESIKSISLEAYNWIMQNSQP
EHWANAVFPGGRYNHMTSNFGELFYSWVSDAHELPITQMVDVIRARIMELIFSRRADSDQ
WARRLTPSMEEKLEKENLKVRSLQVLVPAGNIFEVRGDSIEVVDMDRWECSCKGWQVSGL
PCCHAIAVIGCIGRSAYDFCSRYFTIESYRLTYSESIHPIPCLERDLLNESSQAAVTVTP
PPARRPPGRPTTKRIGSQEHKSYWWAAEYGMRGERIDMVGTLLVIFRKMYFLLSDTWQVG
MIIMWLFGEGKAFVEK
>Cp7
MGKGKLILICQSGGTFITNDDGSLSYTGGEAEAIDINHETVFDDLKFKLAEIWDLDYTSL
SIKYFLPGNRRTLINLSNDRDLKRMYDFHGESVTADVFVTGRKGFDQVAFDMHHNRACGI
KVAETVIATVASQPAMTMPVAMETETGTIDDTSKLAPVASRDNLVAAKKTTKSSTRLTAK
SKRLTTKSSTRVTRTSKRSRAYKIDSDPGAACSVRNATADDCGHGLIIVDMSATPADTVK
KRRRTAYWQSTPNGPTIVSITENVGETRKGTSRRRNVESDSPSAISYYVEQQVETGPLME
DSSGALVLANTGDASSKELIASWKDCITGVGHDFRVWLSFVMHCKNMPLHIVLLSAVKSK
YHEILLTASALDGDDGVFPVAVTLVDIDNVGNWQWFLEQLRSALSTSRSITFVSDREKGL
TKCVLEVFENAHHGYSMFYLMENFMRDLKGPFHGDGRGSLPVNFLAAARAVRVDAFKMFT
ERIKRISPRAYDWVMTVEPEHWADSAFKGERYSQITFNIAELYAKWTQEVWQLPIIQKIE
KLISKMMELINDRRNISSDWSSKLTPSKEEKVEEERRRACDLKVLFSSDTLFEVHDTSIN
VVDIDKRDCSCLAWKVTGLPCRHAIAVFSCTGRSVYDYCSNYFTVDNFHVTYSKSINLFL
SVFNPSDEEKAGSEAETVLPPSISRPPSQEKKKPNKSKGRIKRLVFCTRCKGAGHNKATC
KEIEQGIIRLT
>Vv4
MANHDLILGPNHNLGLGQNQQLVLGHNHNLGLGQNHTLELGQTHEHDLGLGHSHDHELGL
SHAHDHDLGLGQTVHQGADEHGYEHGNELAMDRKPEHDDHGLSLAEQNHELALSENNELA
VSENQELDDNLDLAVDDHQEMGIESTSDMVQQHQLVVSTPVLQARTVVANPSYELVVGQE
FPDVKSCRRALRDTAIALHFEMQTIKSDKTRFTAKCASDGCPWRIHAAKLPGVPTFTIRT
IHEAHTCGGIAHLGHQQASVQWVANSVEQRLRENPNYKPKEILEEIHRVHGITLSYKQAW
RGKERIMAAMRGSFEEGYRLLPQYCDQVKRTNPGSIASVYGNPTDNCFQRLFISFQASIY
GFLNACRPLLGLDRTFLKSKYLGTLLFATGFDGDGALFPLAFGVVDEENDENWMWFLSEL
HNLLEVNTENMPRLTILSDRQKGIVDGVEANFPTAFHGFCMRHLSDSFRKEFNNTMLVNL
LWEAAHALTVIEFEAKILEIEEISQDAAYWIRRIPPRLWATAYFEGTRFGHLTANIVESL
NTWILEASGLPIIQMMECIRRQLMTWFNERRETSMQWTSILVPSAERRVAEALERARTYQ
VLRANEAEFEVISHEGTNIVDIRNRCCLCRGWQLYGLPCAHAVAALLSCRQNVHRFTESC
FTVATYRKTYSQTIHPIPDKTLWKELSEGDPNGSKSVEIMINPPKSLRPPGRPRKKRVRA
EDRGRVKRVVHCSRCNQTGHFRTTCAAPI
>Vv5
MAGKKIIAICQSGGEFEADKDGSLSYRGGDAHAIDIDDQMKFNEFKMEVAEMFNCSISTM
SIKYFLPKNKKTLITISNDKDLKRMIKFHVDSVTVDIYVMTEEVVALDVSNMPASRSSRT
TLSEAVVPVDAPLDMKDDMVDDTTYPDVSLGLPLDVVDDTTHVDVDAQITMPNEISPVLP
LSISNEEKHVKAAQQWQNTITGVGQRFSGVHEFREALRKYAIAHQFAFRYKKNDSHRVTV
KCKAEGCPWRIHASRLSTTQLICIKKMNATHTCEGAVVTTGYQATRSWVASIIMDKLKVF

PNYKPKDIVNDIKQEYGIQLNYFQAWRGKEIAKEQLQGSYKEAYSQLPFFCEKIMETNPG
SFATFTTKEDSSFHRLFVSFHASLYGFQQGCRPLLFLDSISLKSKYQGTLLAATAADGDD
GVFPVAFSVVDAETDDNWHWFLLQLKSALPTSRPITFVADREKGLRESIAEIFQGSFHGY
CLRYLTEQLLKDLKGQFSHEVKRLMVEDFYAAAYAPRPESFQRCLETIKSISLEAYNWLI
QSEPMNWANAFFQSARYNHMASNFGELFYSWASEAHELPITQMVDVIRGKIMELFFTRRT
DSNQWMTRLTPSMEEKLEKETVKVRPLQVLLSGGNTFEVRGDTIEVVDIDHWDCSCKGWQ
LTGLPCCHAIAVISCIGQSPYEYCSRYFTTESYRLTYSESVHPIPNVDRPMEKDSSLVAV
TVTPPPTRRPPGRPTTKRFGSQEVVKRQLQCSRCKGVGHNKSTCKELL
>Vv6
MSNNDLLLGNDHDLSLGHNQPLGLRHNHNLVLSHELVLGHAHDDELALGQNHEHEMALRH
AHGHHNHENGFDRVDENGLDMSQNHDPDVDQHHNDVDNHDNELGLTVQNHALSLSENHEL
ALVENHDLDENIELTVSQSGEISIVDASGMTAQHSQLLVSSPVLQSRTVVPAPNHELVVG
QEFSDVQSCRRALRDTAIALHFEIQTVKSDKTRFTAKCASDGCPWRIHCAKLPGVPTFTI
RTIHESHTCGGITHLGHQQASVQWVASSVEQSLKENPHYKPKEILEEIHRVHGITLSYKQ
AWRGKERIMAAVRGSFEEGYRLLPQYCDQIRRTNPESIALVYANPMDNSFHRLFVSFQAS
IYGFLNACRPLIGLDRTLLKSKYLGTLLFATGFDGDGALFPLAFGVVDEENDDNWMWFLS
ELHNLLEINTENMPRLTILSDRQKVIVEGVEANFPTAFHGFCMRHLSDSFRKEFNNTLLV
NLLWEAAQVLTVIEFEAKILEIEEISQEAAYWIRRIPPRLWATAYFEGTRFGHLTANVVE
SLNTWILEASGLPIIQMMECIRRQLMTWFNERRETSMQWTSILVPSAERRVSEALERART
YQVLRANEAEFEVISHEGTNIVDIRNRCCLCRGWQLHGLPCAHAVAALLSCRQNVHRYTE
SCFTVATYRKAYSQTIHPIPDKTLWKEMADGSQDGGDNAVETIINPPKSLRPQGRPRKRR
VRAEDRGRVKRVVHCSRCNQTGHFRTTCAAPI
>Vv9
MADHALVVSDASLDHSLVVSDASVDHSLVIGQEFPDVETCRRTLKDIAIALHFDLRIVKS
DRSRFIAKCSKEGCPWRVHVAKCPGVPTFSIRTLHGVHTCEGVRNCHHQQASIGWVARSV
EQRVRDNPQYKPKEILQDIRDQHGVAVSYMQAWRGKERSMAALHGTFEEGYRLLPGYCEQ
IRKTNPGSIASVFATGQENCFQRLFISFRASIYGFINACRPLLELDKAHLKGKYLGTLLC
AAAVDADDALFPLAIAIVDTESDENWMWFMSELRKLLGVNTDNMPRLTILSERQSGIVEA
VETHFPSASHGFCLRFISENFRDTFKNTKLVNIFWNAVYALTAVEFESKITEMIEISQDV
IPWFKGFPPQLWAVAYFDGVRYGHFSLGVTELLYKWALECHELPIVQMMEHIRLQLTSWF
DERRNMGMRWTSILVPSAEKRILEAIADAHCYQVLRANEIEFEIVSTERTNIVDIRSRVC
SCRRWQLYGLPCAHAAAALISCGQNAHLFAEPCFTVASYRDTYSQMINPVPDKRHWKEPG
EGAEGGGAKVDITIRPPKTRRPPGRPKKKVLRVENFKRPKRVVQCGRCHMLGHSQKKCTM
QM
>Vv10
MGDYNFVVGQEFADVKAFRNAIKEAAIAQHFELRIIKSDLIRYFAKCATEGCPWRIRAVK
LPNAPTFTIRSLEGTHTCGKNAQNGHHQASVDWIVSFIEERLRDNINYKPKDILHDIHKQ
YGITIPYKQAWRAKERGLAAIYGSSEEGYCLLPAYCEEIKRANPGSVAEVFTSGADNRFQ
RLFVSFYASIYGFLNGCLPIVGLGGIQLKSKYLGTLLSATSFDADGGLFPLAFGVVDAEN
DESWMWFLSELRKALEMNTENVPQLTFLSDGQKGIQDAVKRKFPTSSHAFCMRHLSESIG
KEFKNSRLVHLLWKAAYATTTIAFKEKMAEIEEVSSEAAKWIQQFPTSRWALVYFEGTRY
GHLSSNIEEFNKWILEARELPIIQVIEQIHSKLMAEFEERRLKSNSWFSVLAPSADKRMM
EAIGRASTYQVLRSDEVEFEVLSAERSDIVNIGTQCCSCRDWQLYGIPCSHAVAALLSCR
KDVYAFTEKCFTVASYRAAYSEEIRPIPCKIEWRRTVEAPVDDDTPVVRPPKFRRPPGRP
EKKRICVEDLNREKHTVHCSRCNQTGHYKTTCKADIMKSIEQF
>Vv11
MAAKKVIAICQSGGEFVTNKDGSLSYNGGEAYAIDVDQQTQLSDFKLEVAEMFNCSIDTM
SIKYFLPDNKKTLITISKDKDLKRMVKFLGDSVTVDIFIMTEEAVPRNQSIMPASRLMLF
RMLTFRVQFGAQLQSSRTTVSEAVVPAVAPVDAVVDMTHAIDKVDMDMANYTHSDNAPVI
SNDDKHQKAAQQWENTITGVDQRFNSFNEFREALHKYSIAHGFAYKYKKNDSHRVTVKCK
SQGCPWRIYASRLSTTQLICIKKMHTTHTCEGAIVKAGYRATRGWVGTIIKEKLKVSPNY
KPKDIADDIKREYGIQLNYSQAWRAKEIAREQLQGSYKEAYSQLPFFCEKIKETNPGSFA
TFETKEDSSFHRLFISFHAAISGFQQGCRPLLFLDSTPLNSKYQGMLLTATAADGDDGVF
PVAFAVVDAETDDNWSWFLLELKSAVSTARPITFVADFQKGLKKSLAEIFDNGYHSYCLR

YLTEKLNKDLKGQFSHEARRFMINDFYAAAYASRLETFQRCTENIKGISPEAYNWVIQSE
PDHWSNAFFGGARYSHMASNFGQLFYNWVSEANDLPITQMVDVLRGKMMELIYKRRVDSS
QWITKLTPSKEEKLLKDTSTARSLQVLLSHGSTFEVRGESIDIVDIDHWDCSCKDWQLSG
LPCCHAIAVFEWIGRNPYDYCSRYFTVESYRLTYAESIHPVPNVDRPVKTESTQVGIIVT
PPPTKRPPGRPKMKQAGSVETIKRQLQCSKCKGLGHNKKTCKDF
>Vv12
MWNLEYQSLSIKYFLPGNRKTLITLSTDKDLKRMIGFHGDSVTADVFVMGREGFDSHALN
IHACRESGIKLAETVNHIAVSMTPAVAPQPFAIAPVSLGVMPSGAFPIDPVTVVTDVASP
DTTTTVAHAAVTVSPVAPATFLVSTVADSLTAVDATAQSLNGISTTANPFAAFTITGDPS
AAAPTPTVPVVIAAIDATAHGSVILDITSTPADTVKKRRRTASWKFGANSPTIVSVTDDV
GGKKRTASRKKNSRSQNTVPVADNVEQQQENGPWKDDFNGSSSLVASDDVPLEKLVASWK
DGITGVGQEFKSVYEFREALQKYAIAHRFVYRLKKNDTNRASGRCVAEGCSWRIHASWVP
AAQSFRIKKMTKSHTCGGQSWKSAHPTKNWLVSIIKDRLQDTPHHKPKDIAKCIFQDFGI
ELNYTQVWRGIEDAREQLQGSYKEAYNLLPWFCEKLVETNPGSVAKLLINDDKRFERLFV
SFHASLHGFQNGCRPLLFLDATSLKSKYQEILLIATAVDGNEGFFPVAFAIVDVETDDNW
LWFLEQLKSAISTLQPMTFVSDREKGLKKSVLEVFENAHHGYSIYYLMENFKKNLKGPFH
GDGRGSLPINFLAATHAIRLDGFKKSTEQIKRVSSKAYNWVMQIEPECWATVSFEGEHYN
QITVDVIHAYINLIEEVRELPIIQKIEALICMIMESINTCQTDSSTWSSQLTPSKEEKLQ
DEIIKARSLKVLFSTDTLFEVHDDSINVVNIDSWDCSCLQWKATGLPCCHAIAVFNCTGR
SVYDYCSRYFTLNSFRLTYSESINPLPSIIKSLDNEEAALHTLNVLPPCTLRPLSQQKRK
RVKTEEVMRRAVSCTRCKLAGHNKATCKATL
>Zm1
MANHELVLGHGQDAELALGPNHHDFGHSHDLDLGGHAHDHELVLGQSHEHDHELVLGQSH
ERDHELVLGQSHEHDDELVLGHHHDDDHQLVLGHDHHHHHDELDLSESHELTLAETHHLG
VDHNLDQLSLEQAHELALQPHDFSQGPLAVAPVVQSRTMVVSPEFQLAVGQEFPDVMSCR
RAIRSTAIACHFEIQTVKSDKTRFTAKCAAEGCPWRIHAAKLAGVPTFSIRTIHDTHSCV
GISHLGHQQASVQWVATTVEERLRENPQCKPKEILEEIHKAHGITLSYKQAWRGKERIMA
AVRGSFEEGYRLLPEYCRQVERTNPGSIARVYGNPDDSCFRRLFISFNASIYGFINACRP
LIGLDRTLLKNKYLGTLFLATGFDGDGDLFPLAFGVVDEETDENWVWFLSELHELLEKNT
ENMPRLTILSDRQKGITDGVEFNFPTAFHGYCMRHVSETFKKEFSNPVLVNLLWEAAHAL
TVIEFETKLLEIEDMSQEAAVWIRHLPPRLWATAYFEGTRYGHLTASITESLSSWILDAA
GLPVTQMMECIRRQLMTWFNERREASMQWSTILVPAAERRVQEAIERARGYQVARANEAE
FEVVSAHEGTNIVDIRNRCCLCRGWQLYGVPCAHGVAALLSCRQNVHRYAESCFTVATYR
KTYSQTIHPIPDKTLWSETADQDQGQDGEVKAEMVINPPKSLRPPGRPRKKRVRAEDRGR
VKRVVHCSRCNQTGHFRTTCAVPI
>Zm2
MANHELVLGHGQDAELALGPNHHDFGQDHGLGLGHSHDLGLGGHAHDHELVLGQSHEHDH
QLVLGHHHDDDHQLVLGHDDHHHHHVHHAGELVLRQGHDGDSETLEVQGHHHDELDLSES
HELTLAETHHLGVDQNFDQLSLEQAHELALQPHDLSQAPLAVAPVVQSRTMVVSPEFQLA
VGQQFPDVVSCRRAIRNTAIACHFEIQTVKSDKSRFTAKCAAESCPWRIHAAKLPGVPTF
SIRTIHDNHSCVGINHLGHQQASVQWVANTVEERLRENPEYKPKEILEEIHKAHGITLSY
KQAWRGKERIMAAVRGSFEEGYRLLPEYCRQVERTNPGSIARVYGNPDDNCFRRLFISFS
ASIFGFVNACRPLIGLDRTLLKNKYLGTLFLATGFDGDGALFPLAFGVVDEETDENWVWF
LSELHELLEKNTENMPRLTILSDRRKGITDGVEFNFPTAFHGYCMRHVSEAFKKEFNNPV
LANLLWEAAHALTVIEFETKLLEIEDASPEAVVWIRHLPSRLWATAYFEGTRYGHLTANI
AESLNSWILDASGLPIVQMMECIRRQLMTWFNERREASMQWNTILVPAAERRVQEAIERA
RGYQVARANEAEFEVISAHEGTNVVDIRNRCCLCRGWQLYGVPCAHGVAALLSCRQNVHR
YTESCFTVATYRKTYSQTIHPIPDRTLWDETADQGQVKVEMIINPPKSLRPAAQPRKKRV
RAEDRGRVKRVVHCSRCNQTGHFRTTCAAPI
>Zm3
MEDNQILVQSEHNLIPGSRTLVIGQEFADVDTCRRAVKDMAIALHYELRVVKSDRSRFIA
KCSKEGCPWRVHIAKCPGVPTFTVRTLHGEHKCEGVLNLHHQQATVGWVARSVEARLRDN
PQIKPKEILQDIREQHGVAVSYMQAWRGKERSMAAVNGTLEDGYRLLPAYCEQIVKTNPG
SVATYRGIGPGNAFQRLFVSFRASIYGFLNGCRPLLEIDKADLKGKYLGTLLCASAIDAD

HMMFPLAFGVVDCESDDNWNWFISELRKMLGVNTDKMPVLTIVSERKRQVVAAVGSNFPT
AFHGFCLRHVSENFRDEFKNTKLLNIFWSAVYALTASEFDAKVQDVMPWLQRFPPNLWAF
SYFQGIRYGHFSLGITEILYNLSLDCHELPIVQTIEHIRHQLACWFAERQNLAQSYNSVL
VPSAEKVILEAIHDSQCYQVLRANKVEFEIVSSERTNIVDTQARFCSCRRWQIYGIPCAH
AAAALLSCGEDPRLYADDCFSVMKYRETYSQPIYPIPDRSHWNDSSPWSQGAVSKADVVL
GPPNIRRPPGRPKMKILKIESMKRPKRIVQCGRCHLLGHSQKKCSLRS
>Zm4
MEGNQILVQSEQSLIPGVQTLVIGQEFADVDTCRRAVKDMAIALHYELRVVKSDRSRFIA
KCSKEGCPWRVHIAKCPGVPTFTVRTLHGDHKCEGVLNLHHQQATVGWVARSVEARLRDN
PQIKPKEILQDIREQHGVAVSYMQAWRGKERSMAAVNGTLEDGYRLLPAYCEQIVKTNPG
SVATYGGIGPGNAFQRLFVSFHASIYGFLNGCRPLLEIDKADLKGKYLGTLLCASAIDAD
HMMFPLAFGVVDSESDDNWNWFISELRKMLGVNTDKMPVLTILSERKRQVVKAVGSNFPT
AFHGFCLRYVSESFLDEFKNTKLLNLFWSAVYALTASEFDAKVNEMMRVQDVMPWLQRFP
PNLWAVSCFQGIRYGHFSLGITEILYNLSLDCHELPIVQAIEHIRHQLTCWFAERQNLAQ
SYNSVLVPSAEKLVSEAIHDSQCYQVLRANKVEFEIVSSERTNIVDTEARSCSCRRWQIY
GIPCAHATAALLSCGEDPRFYAHDCFSVMKYRETYSQPIHPIPDRSHWSNSSPGLQGVAS
KADVMMLSPPNIRRPPGRPKMKILKIESMKRPKRIVQCGRCHLVGHSQKKCSLRSCDSYP
H
>Zm5
MAAAAATPPWASLGPDTCFFHYCCHHIFEIRVFQLEFGSDVAFATPAGGASTAPDILDQQ
NLALVDTAHREPIGHFDDSGDSYVGSEITVDPQHGLDNPVMFWDDIIKGVGQEFDNVKDF
RAQLCKYAIGKGFAYRFIKNETTRVTVKCVAEGCTWRMHASESSRNKKFVIKKMTDEHTC
GGGNGEGQRRATRQWLTTVIKEKLHDNPMLKPKDLVKEIYEQYGVTLTYSQVWRGKEVAQ
KELYHAIRETYSHLPWYCERLTESNPGSIALLSPMVDTKFRRFFVAFHASLHGFANGCRP
LLFLDKVPLKATNEYKLLVAAAVDADDAVFPVAFSVVEDENYESWVWFLMQLKFPVQNHS
YAYNAMTFLSSGQKGLDAAVSQVFEDSHHAFCLHHIMEEFKGELRKGPWSQQIREGMIED
FTRAAQACSIEDFNTSIESIRNISTEAAEWIIASKPEHWSDAIFRGCRYDHFSSNIVDAF
NNWIPTKKEGSIVLMVDSLRTKIMEIIETRREACKAWSGPLTPSMEFKAQEEVTKAGKMT
VLCSSETVFEVRGNAIFVVNLANWECTCRRWQLSGLPCLHAVAVFNRIGRSFYDYCSKFF
RIESYHMTYSGTIFPIPDMDTVDFSAGANLIPPPKPRTSDKPRRKRFNPNKIPTVIRLCS
RCKQAGHNKATCEAIL
>Zm6
MGKGQILAVLQIGGEFNTDEDGHMSYSGGEAHAMHVQSDWTFSAFKQEISSTLNNLKLDQ
FAFKYFLPKNDKTLISISNDKDLRRMVEFHAESDSTYIYVMKKADNRSKNIVAVLDTPTD
AFAIVPTTQDGSKRQKVCASWKNVITGVGQVFEGPKDFRDALHKYAIAHRFHYRFIKNDS
SRVTAECTGEGCPWRIHASKSPAKKEFMVKKISESHTCESETVKSNRLASQRWVASVIKE
KLRDSPNYRPRDIANDLQREYGLCLNYSQAWRGRSIAQKELYSTHEEACSQLPWFCERIV
ETNPGSVATVVALEDSKFRFFVAFHASLHGFEHGCRPLLFLEAITAKPNKHWKLLAAASV
DGEGDVFPVAFGVVDDESRENWHWFLEQLKSSLGTSRTITFISNGEHGLWDVLSSVFQEC
HHGYCVESLIEEFKMQLDDAWTEELKDSMVEHLKKAINSCTADEFNQYIELIKSGSDKLA
DWLLEIKPERWSDAFFKGSRHGQYSCNIFGTVSEWIPTRYELSVVQLVDTIRCKLMEMMY
TRRESSNAWTEVLTPAANQKLQEEVNKAHTLNVLPAENGENGNVFKVCDDSVNVVNLDTW
ECTCQRWHISGLPCMHVIAVLERTGQYAYDYCVKYFTTECYRLTYSLSINPIPDVIVPPT
LIDLAQSPATYPCPLRTRRRVGRPKEKPADPRIAIKRAVRCSRCKGYGHNKATCKVPISS
FST
>Zm8
MADGAIVVAICQYGGEFTSGPSGNLIYRGGEAHAVDVTHDSSLESFKDELSKVFHVDVTD
MSLKYFLPNNMKTLITISCDRDLQRMVGFTANAAHVDVFLISRQENRSTITHSGATSGSV
ASGDKRKRPTPKNKVIRSSKPATSAACNALQAITDNVRQPISVITENEDNRVFQLEFGND
ITFTTTAGGASFTSDILDQQKLVLVDNIPKEAVSLFDDAFNPYVSSEIMHEPPQEPNNPI
VLWDDIIKGVGQEFDNVKDFRAQLCKYAIGKGFVYRFIKNETTRVTVKCGAEGGCTWRLH
ASESSRNKKFIIKRLTDEHTCGGGSGEGQRRATRQWLTTIIKEKLHDNPKFKPKELVKEL
FEEYGVTLTYSQVWRGKEVAEKELYHAVRETRDQLPWYCQRLEETNPGSISVLSPVLDMK
TRRFFVALHACLHGFINGCRPLLFLDKVPLKATNEYKLLVAAAVDADDGVFPLAFNVVED

ENYDSWGWFLMHLKIALQTHNYPCNGMTFLSNGQMGLDAAVSHVFEDGEHAFCLHHIIEE
FKGELRKGPWSQQIREGMVEDFTRAAQACSIEDFNASIESIRNISSEAADWIIASKPEHW
SDAIFRGFRYDHFSLNIVDAFDNWIPAKKESSIVLMVDSLRIKIKEVIESRLEACKVWQG
PLTPTMEYKVQDEMLKAGKMTVLCSSDTVFEVRGNAVFVVNIGNWECTCRRWQLSGLPCM
HAIAVFNRLGRCFYDYCPKFFRIESYHLTYSGIIFPIPDLDSVDFSAGANLLPLPPKRRT
SDKPRRKRSNPNKISTLTRLCSRCKQTGHNKATCEVQF
>Bd1
MANHELVLGHGQDAAELALGQNHHDFGQDHHGLVLGHSHDLGLGHPQDHDFVLSHDHGHE
HDHALVLRHHHHHDSQLVLGHDHTGDLALGQGHDEDSLDGEGHHDLGLSENHELALAEPH
PLVDDDNLDHHFTLEQAHELALEPAHDFSQGPLAVAPVVQSRMMVVSTEFQLAVGQEFPD
VMSCRRAIRDTAIACHFEIQTVKSDKTRFTAKCAAEGCPWRIHAAKLPGVPTFSIRTILD
NHSCVGINHLGHQQASVQWVASTVEERLRENPHCKPKEILEEIHKSHGITLSYKQAWRGK
ERIMATVRGSFEEGYRLLPEYCRQIERTNPGSIARIYGNPDDNCFRRLFISFYASIYGFV
NACRPLIGLDKTTLKNKYLGTLFLATAFDGDGALFPLAFGVVDDENDENWIWFLSELHEL
LEKNTENMPRLTILSDRRKGIVDGVDINFPTAFHGYCMRHLSETFRKEFNNSVLVNLLWE
AASVLTATDFETKLLEIEDTSQEAVAWIREIPPRLWATAFFDGPRYGQLTANITESLNSW
ILDASSLPINQMMECLRRQLMTWFNERREASMQWTTILVPTAERRVQEAIERAQNYRVAR
ASEAEFEVISPHEGTNVVDIRNRVCLCRGWQLYGMPCAHGVAALLSCRQNVHRYTESFFT
IAMYRKTYSQTIHPIPDKTHWNETPEDQGEAEQGRLEVIINPPKSLRPPGRPRKKRVRAE
DLGRVKRVVHCSRCNQTGHFRTTCVAPI
>Bd2
MVLGNDHHGHTCDLSLRQGHDKDSLVREGQDGEGHHGFGLLENHELVLAEPHSLVDDDNL
DELSLEQTHEFALQPAHDLSQGLLAVAPRMMAFSTEFQLVAGQEFPNVMYCRRAIRNTAI
ACHFEIQMVKSDNTSFTAKCTAEGCPWHIHAAKLPGVPTFSIRTILDNHSCVGINHLGHQ
QASVQLVASTVGERLPENPHCKPKEILEEIHKSNEITLPYKQDWIGKERSIAAVRGSFEE
GYRLLPEYCRQVERTNPGSIGRVYGNPDDNRFRRLFISFYASIYGFVNACCPLIGLDKVT
LKNKYVGTLFIATAFDGDGALFPLAFGVVDEETDENWIWFLSELHELLEKNTLSMPRLTI
LSDRSKGIIDGVDINFPAAFHGYCMHHLSETFRKEFNNSVPVDLLWEAANVLTAIDFETK
LLEIEDISQEAVCWIKGIRPCLWATAFFDGTRYGHLTANVTESLNSWILDASSLPINQMM
ECLRCQLMTWFKERHEASMQWTATLVPTAERRLQEAIERARGYQVTRANETEFEVISPHE
GTNVVDVRNRCCLCRGWQLYGVPCAHGVAALVSCRQNVHRYTERYFTIGTYRKTYSQTVH
PIPDKTLWNKMSNQGEAEESKLEVIINPPKSRRSPGRPRKKRVHAEDHGQVKLFVHCSRC
SQMGHFRTTCPAAL
>Bd3
MEGNKVTVEPADGQVMVEREDDQVSVELEEDQVLVETDDDQALVEPEQDLALREQFLVIG
QEFANVDACRRAIKDMAIALHFQLRVVKSDRSRFIAKCSSEGCPWRVHVAKCHGVPTFTV
RTLHGEHTCEGVQDLHHQQATVNWVARSVEARLRDNPQIKPKEILQDIRDQHGVAVSYMQ
AWRGKERSMAAVHGTLEDGYRFLPSYCEQIVKTNPGSVAVYKGSGPENSFQRLFVSFRAS
IYGFLNGCRPLLEIDKSDLKGKYLGTLLCASAVDADHMMFPVAFGVVDSESDENWMWFVS
ELRKMLGVNTDKMPVLTILSERQTQVVEAVEVNFPTAFHGFCLRYVSENFREEFKSPKLL
NLFWSAVYALTTAEFDSKVKDMMHIQDVMPWFEHFPPNLWAVAYFDGIRYGHFNLGITEI
LYNWALECHELPLVQTVEYIRNQLTCWFTERDKLALSLNSVLVPSAEKLISEAIADSRCY
QVLRANKVEFEIVSSERTNIVDTQTRFCSCRRWQIYGIPCAHAVAALLSCGEDPRLYAHE
CFSVTKYLETYSQRIHPIPDRSQWSSSCSGLRGPGYKSDAILRPPRIRRPPGRPKMKILK
MESLKRPKRIVQCGRCHLLGHSQKKCSL
>Bd4
MVALVGDGRRGNRTLMGKGQIVAVLQLGGEFTTDADGHMSYSGGEAHAMLVKSDWTFDAF
KHEISSTLNNLKIDQFLFKYFLPKNNKTLISISNDKDLLRMVEFHAESETTYIYVMKKVD
NRAKGVVADSGTPVETTAVVPTTEDGSKRQKVCATWKNVITGVGQVFEGPKDFRDALHKY
AIAHRFHYRFVKNDSSRVTAECTGEDCPWRIHASKSPAKQDFMIKKISESHTCESETVKS
HRLASQRWVASVIKEKLRDSPNYRPRDIANDLQQEYGLCLNYSQAWRGRSIAQKQLYSSH
DEACNQLPWFCERIKETNPGSVATVVTMEDSKFCFFVAFHASLHGFEHGCRPLIFLDAVS
AKPNKQWKLLAATSVDGEGDVFPVAFTVVDEESRENWHWFLEQLKSSLLASRDITFISNG
ENGLWDEVPLVFPESHHGYCVDFLIEEFKMQLEDAWIEEVRDAMVELCKKAIYSCTADEF

NQHIEEIRSESDKLAEWLLEIKPERWSDAFFKGSRHGQYSSNIYNTIADWIPTRYELSVV
QLVDTIRCKLMELMYTRRESSNEWTEVLTPAANQKLQEEVSKSHTLNVTPTESDGQGSVF
KVCDDSVNVVNIDTCDCTCRKWHVSGLPCMHAVAVFERTGQYAYDYCLKYFTTECYRLTY
SISINPIPDVILPPVTLTNPSQSPATYPCPIRTRRRVGRPKEKPADPRITIKRAVRCSRC
KGYGHNKATCKIPITEALPLAELSDVQN
>Bd5
MGSRQIVAVLQLGGVFTTDDDGGMTYSGGEAHAMHVKSGWTFKAFKNEISSTLNNLKLDS
YAFKYFLPRNNKTLISISNDKDLKRMVEFHAESETTYIYVIKKVENRVKSSVADSGAPAD
LAVIATTPDGSKRQKICASWENAITGAGQVFEGPKEFRDALHKYAIARRFHYRFIKNDST
RVTVECTDEGCPWRIHASKSPSKKEFMIKKVVGSHTCASETVNSHRLASQKWVASVIKEK
LRDSPNYRPRDIANDLQREYGLCLKYSQAWRGKLIARKELYSPHEEACNHLPWFRDRILA
TNPGSLATVVTLEDSKFRFFVAFHASLHGFEHGCRPLIFLDVISVKPNKHWKLLAATSVD
GEGDVFPVAFSVVDDECQENWHWFLEQLKASLPVPREITFISNGKSDLWDDVSLIFPDSY
HGYNINSFIEEFKTQLDDGWSEEVKDTMVEHLKEAVYSCTVDEFNHYIELIKAESDKLAE
WLMETKPERWSDAFFKGSRLGQYTSNISETIVDWMPSRYELPVVQLLDTIRCNLMEMIYT
RRESSNAWSEVLTPSANQKIQEEMSKVLSLSVVCSTENHGNNNVFEVCDGSVYIVNIDTW
ECTCRKWHVSGIPCSHALAVFERTEQNPLDYCAKYFTTECYRMTYAMSINPIPDVMVPSA
STDPSQSGGLVPCPILTRRQVGRPKEKPADPRIAIKRAVRCSRCKGYGHNKATCKVPITA
>Bd6
MAEGGIVVAICQYGGEFTSGPNGLIYKGGEAHAVDVSREMSLDSFKDEVSKVFHVDVTDM
SFKYFLPNNNKTLITISCDRDLQRMVEFTASAAQADVFVISRVENRASKSSKKTPDASGA
AVQASTVDVNEPRPVLTLNDYNEYRTNLYIIAHLYMYIVPLVLLPISCRDFPLEFGREAA
FDTAAGAISSAPDTLNQEKLALVDNTQREPIGFFDDAINPYDGSEIIIDPPQELNNNPTV
FWDDIIKGVGQEFDNVKDFRAQLCKYAIGKGFVYRFIKNETTRVTVKCVGDGCTWRLHAS
ESSRNKKFVIKKMTDEHTCGGGGGEGQRRATRQWLTTIIKEKLHANSLLKPKDLVKEIHE
EYGVLLTYSQVWRGREVAQKEMFHVMTETLGHLPWYRDKLLQTNPGSILDLPGNRRFFIA
FHASLHGFTNGCRPLLFLDKVPLKATNEYKLLVAAAVDADDGFFPVAFNVVEDENYESWV
WFLTKLRIALQYHNYPLNDMTFLSNGQKGLDAAVPHAFEDSHHAFCLHHIMEEFKGELRK
GPWSQQIRDVMVEDFTRAAQACNTDDFNASIESIRNISTEAADWIIASKPEHWSDAVFGG
CRYDHFSSNIVDAFNNWIPTKKEGSVVLMMDSLRIKIMETIEARREACKSWEGPLTPSMD
YKAQDEMSKAGKLTVLCSSETVFEVRGSGISVVNLANWECTCRRWQLSGLPCMHAFAVFN
RIGISFYDYCSKFFRIESYHSTYSGTIFPIPDMDTFDFSAGATIPPPKPRTSDKPRKKRF
NPNKIRVHAAQIQ
>Bd7
MAEGGIVVAICQYGGEFTSGPNGNLIYKGGEAHAVDVSREMSLDSFKDEVSKVFHVDVTD
MSFKYFLPNNNRTLITISCDKDLQRMVDFTASAAQADVFVISRVENRSIVTYSGALTVKP
GSSANGDKRKRPSPKKKASKSNTNTPTATATAVQANTNDVNQPRPVVTLNDYNGDFQLEF
GHDVALATTAEAVSSAPDVLNQEKLALVDNTPRDPVGLFDDAINAYDGSEIIIDPPQEFN
DNPTVSWDDIIKGVGQEFDNVKDFRAQLCKYAIGKGFVYRFIKNETTRVTVKCVGDGCTW
RLHASESSRNKKFVIKKMTGEHTCGGGDGEGQRRATRQWLTTVIKEKLSKNSLLKPKDLV
NEIYEGYGVLLTYSQVWRGREVAQKEMFHVVRETFGHLPWYKERLLQTNPGSILDLSGLA
DTKFRRFFFAFHASLYGFANGCRPLLFLDKVPLKATNEYKLLVAAAVDADDGVFPVAFNV
VEDENFDSWVSFLTNLRFALEHHNYPLNVMTFLSNGQKGLDAAVPHVFEGSHHAFCLHHI
MEEFKGELKKGPWSQQIRDAMVEDFTRAAQACSIDDFNASIESIRNISTEAADWIIASKP
EHWSDAIFAGCRYDHFSSNIVDAFNNWIPTKKEGSMVLMMDSLRIKITETIEARREACMS
WSGPLTPSMDYKAQDEMAKAGKLIVLCSSETVFEVRGSGIFVVNLANWECTCRRWQLSGL
PCMHAVAVFNRIGRSFYDYCSKFFRIESYHLTYSGTIFPIPDMDSFDFTAGATIPPPKPR
TSDKPRRKRINPNKTTTLIRLCSRCKQVGHNKATCEAIL
>Mg1
MSNHNNVLLGQNNAIPLNRIQHVVLNRNHNMVIAQNHVLEIGQTHEQETELVQTHDQQVD
NGDEFEQQNGLSIEEKPDQEELDISDQDELAISETQELDENMELAIHHEDMTMDSMHDDD
EMGLHHALVLTPSHVIQQRTLALTPNFEIHVGQEFPDVRACRRALRDTAIALHFEMQTIK
SDKTRFTAKCANESCPWRIHAAKLPGVPTFTIRTINDSHTCGGIGHLGHQQASVQWVANS
VEQRLRENPNCKPKEILEEIHRVHGITLSYKQAWRGKERIMSAMRGSFEEGYRLLPQYCE

QIKRTNPGSIASVYGNPADNCFKRLFISFQASMYGFLNACRPLLGLDRTFLKSKYLGTLL
LATGFDGDGALFPLAFGVVDEESDENWMWFLSELHNLLEVNTENMPRLTILSDRAKGIVN
GVEANFPTAFHGFCMRHLSESFRKEFNNTMLVNLLWEAAHALTVIEFDNKILEIEEVSQD
AAYWIRRLPARLWATAYFEGTRFGHLTANIVESQNSWILEDSGLPIIQMMECIRRQLMTW
FNERRETSMQWTSILVPTAERRVAEALERARTYQVLRANEAEFEVISHEGTNIVDIKNRC
CLCRGWQLYGLPCAHAVAALLSCRQNVHRFTESCFTVATYRKTYSQTIHPIPDKTLWTEL
SEGDVDKVSEMVIHPPKSLRPPGRPRKKRVRAEDRGRTKRVVHCSRCNQTGHFRTTCAAP
I
>Mg2
MVRREICHWLRTRTSASSTVITPPIQQCRSFPPLAHNHEPDINLRSLDDSELGLEHDINH
EDYFLAPPIDNELDIPHVQDGEYHNEYDQGSDHENKYELDDQKTLDVESIETKINEDDSV
TDRQLSLASQSNELGFVENNNLSLTEHGEVDSNLELSAHQNPDMVSSHLSVLQPGYVITS
PIVQSRTVIPAPVQELTVGQEFPDVKSCRRALRDMAIALHFEIQTVKSDKTRFTAKCASD
GCPWRIHAAKLPGVPTFTIRTIHSEHNCGGIAHLGHQQASVQWVASSVEQRLRENPHCKP
KEILEEIHRVHGITLSYKQAWRGKERFMASFRGSFEEDYRLLPQYCNQIRRTNPGSMASV
CVNPVDNSFLRLFISYQASIYGFLNACRPLIGLNRTVLKNKYLGALLFATGFDGDGELFP
LAFGVVDEENDETWMWFLSQLHNLLETNTGNMPRLTILSDRRKCIVDAVESNFPTAFHGL
CMLHLSENFRKEFNSISLCNLLWEAANAPTESEFSAKICQIAETSQDAAYWLQQQFEPRL
WASSYFEGTRFGTLTADVVGSLNSWILEASGMPIVQMMESVRRQLMTWFNERREASMQWN
SVLVPSAEQRVAEAVECSRMFQVTRANDMEFNVITGKGNQVVDIRRWLCSCRGWQLYGVP
CSHAAAALIFCKQNVHRFAEGCFTVATYRKTYSQTIHPIPDRTVWHEMCGGGGEEEEEIV
VVNPPKSLRPPGQPRKRRAAQSGDRSGGQAAKRVVHCSRCYQTGHFRTTCATPI
>Mg3
MEGHDFIIGQEFPDVKAFRNAVKETAIAQHFELRILKSDLIRYIAKCAAEGCPWRIRAVK
LPNAPTFTIRSLDGTHTCGKNAHTGHHQASVDWIVNFIEERLRENVNYKPKDILRDVYEQ
YGITIPYKQAWRAKERGLQSIYGSSEEGYFLLPLYCEQIKKNNPKSIAEVFTSGSDNRFQ
RVFISFYASIKGFLNGCLPIIGLGGIQLKSKYLGTLLSATSFDGDGGLFPLAFGVVDVES
DESWMWFLSELRKALEMNTEIVPQITFLSKWQNGVGDAAKRKFPTSCHAVCMKHLTESIV
REFKNPRLVQLLWKATYATSNIGFKEKMVEIEEISSEARNWLQQNYPPSRWAMMYFDGAR
FGHLSSNTDEFNQWVLEARELPITQVIEQIQTKLMVEFEERRAKSKTWFSVLTPSAEKHV
IEAVNLASTYQVLRSDEVEFEVLSAERSDIVNIGSNSCSCCGWEMCGLPCSHGVAALLFS
RKDVYAFTGNKYFTTASYCAAYEEEIHCVPGKTELIKEGETTLDGEIRSVRPPKVKRPPG
RPEKKRMCIEDFNREKHTVHCSRCNQTGHYKSTCKSDGCKSVLAQI
>Mg4
MAEHDSSTAPNANVDATVTLAVQLAENVFPDSGVDEGLSLVSHTLSIGQEFPDVDTCRRT
LKDIAIALHFEIRIVKSDRSRFIAKCSKDGCPWRVHVAKCPGVPTFTVRTLHAEHTCEGV
QNLHHQQASVGWVARSVEARVRDNPQYKPKEILQDIRDQHGVAVSYMQAWRGKERSMAAL
HGTYEEGFKLLPAYCEQIKKTNPGSIASIVATGQENSFQRLFVSFRAAIYGFINACRPLL
ELDRVNLKGKYLGTLFCAAAIDADDALFPLAVAIVDVESDENWMWFMSELRKLLGVNTDA
MPRLTILSERTMGMAEAVETHFPNAFHGFCLRYISENFRDTFKNSKLVNIFWNAVYALTT
AEFESKISEMVQISEDVLPWFHHFNPQLWALAYFEGVRYGHFTLTVTELLYNWALECHEL
PIVQMVEHIRHQLSSWFNDRRNLGMRLTSILVPSAEKRIAEAISDSHCYKVLRANEVEFE
IVSTERTNIVDISTRVCSCRRWQLYGLPCAHAAAALISCGQNAHLFAEHCFTVHSYRETY
SQMIYSIPDKSMWKENGEGIEGGGAKVEITIRPPKTRRPPGRPKKKVLRMESLKRPKRVV
QCGRCHMLGHSQKKCTLPM
>Mg5
MATKKIIAICQSGGEFETNKDDGSLFYTGGEAYALDLDHKTQLKDFKRELAETFQFRAAA
LSIKYFLPGNRKTLITISKDKDLKRMVNFFKDTDQVEVFVVAEEVDVAAPNVSNMPASSQ
SGLPVSDAHVLYILDFTALMLLNLLIHVVIFFNASKIFSTPLAHLTYPFIVFVLSHLKET
ETSCFVIKLVFLRLIIMKSSRSSRTTVSAAAVPSDVPVDVMQTDDAIVLDEPIETTPLGA
CSFSNEDRHRRAATQWENIITGVDQRFNTFAEFREALHKYSIAHGFTYKYKKNDSHRVTA
KCKTEGCPWRIYASRLATTQLICIKKMNPEHTCEGATVKAGYRATRGWIGNIIKEKLKVS
PNYKPKDIASDIKREYGIQLNYTQAWRAKEIAREQLQGSYKEAYSQLPFFCQNIMETNPG
SLATFSTKEDSSFRRFFVSFHASISGFHQCRPLLFLDSTLLYSKYQGTLLAATAADGNDD

FFPVAFAVVDEETEDNWNWFLTELKSALSTSEQITFVSDFQKGIKNSLIEIFGNECYHGY
CLRSLAEKLNKDLKGQFSHDARRLMVQDFYAAAYAPKIEVFERCAENIKAISIEAYDWVI
RSEPEHWANAIFGGARYNHMTSNFGQQFYGWVSEVDELPITQMVDVLRGKIMELIYRRRL
ESSQWVTRLTPFMEDKLQLEMSKSRSFQVALAHTSTFEVRGGESVDIVDIDHWDCSCKGW
QLSGLPCCHAIAVILCLGRSLYEYCSRFFMTESYRLTYTESINPIPNVEKPERSELHEAT
IVTPPPTKRPPGRPKLKSAESADVIKRQLQCTVRELRVNGVGVEEPETPILTVLGTEEPE
GSS
>Mg6
MATKKIIAICQSGGEFVANKDDGSLFYTGGEAYALDLDHQTQLKDFKHELAETFQFSARA
LSIKYFLPGNRKTLITISKDKDLKRMVNFFKDTDQVEVFIIAEEEEAAAPNVSNMPASRS
SRTTVSGAAVPSDVPVDVMQTDDAIVLDEPISNEDRHRRAATQFNTFAEFREALHKYSIA
HGFTYKYKKNDSHRVTAKCKTEGCPWRIYASRLATTQLICIKKMNPEHTCEGATVKAGYR
ATRGWIGNIIKEKLKVSPNYKPKDIASDIKREYGIQLNYTQAWRAKEIAREQLQGSYKEA
YSQLPFFCQNIMETNPGSLATFSTKEDSSFRRFFVSFHASISGFHQCRPLLFLDSTLLYS
KYQGTLLAATAADGNDDFFPVAFAVVDEETEDNWKWFLSELKSALSTSEHITFVSDFQKG
IKQSLIDIFGNECNHGYCLRSLAEKLNKDLKGQFSHDARRLMVQDFYAAAYAPKVEVFER
CAENIKAISIEAYDWVIRSQPEHWANAIFGGARYNHMTSNFGQQFYSWVSEVDELPITQM
VDVLRGKIMELIYRRRLESSQWVTRLTPFMEDKLQLEMSKARSFQVALVHGSTFEVRGGE
SVDIVDIDHWDCSCKGWQLMGLPCCHAIAVINCLGRSLYDFCSRFFTTESYRLTYTESIN
PIPNVEKPERSELHEATIVTPPPTKRPPGRPKLKSAESVDVIKRQLQCSKCKGLGHNKKT
CYKVNGVGVEEPETPTLTVLGTDEPEGSS
>Mg7
MVVKKIITICQSGGEFETDADGVISYKGGDAHAMEIDDKLKFKDLKSEVAEMFGCNLGTV
AVKYFLPGNKKTLISISNDKDLKRMIKFHNDSDTAEIYVVTEEVAAPDVSQMPGSRSSRT
TLSEAGVGVPVEPSRSTINNIVDDINEPDPLVDSTFDVVGDTNVSTEVPVPSDFPTPIAF
ASPYDEKLAKAAQQWQNNITGVGQRFNSVHEFREALRKYAIAHQFAFKYKKNDSHRVTVK
CKAEGCQWRVHASRLSTTPLICIKKMNPTHTCEGSVLATGYQATRSWVASIIKEKLKVFP
NYKPKDIVTDIKEEYGVQLNYFQAWRGKEIAKEQLQGSYKEAYSQLPYLCEKIMETNPGS
LASFATKEDSSFHHLFVSFRASLYGFEQGCRPLLFLDSIFLKSKYQGSLLAATAADADDG
FFPVAFAIVNVESDENWKWFLQQLKTALSTTPRGLTFVADREKGLKESIAEIFQGQEVYH
AYCLRYLSEQLIRDLKGQFSHEVKRIIIQDMFTAAHAPTEQIFYKCVDSIKAISVTAYNW
IMQSDPTHWANAFFQGARYNHMTSNFGELFYSWVSDAHELPITQLVDAIRTKIMELVYSR
HMEALDWMRRLTPSMEKKLEKENLNVRALEVVSLGDRIEVRFENSVQIVNVDRWECSCRV
WQLTGLPCCHAIAVIMCLQRDPYDFCSRYFTTESYRLTYAETLNPISNSDGPMWQKGEST
QIVTVTPPPTRRPPGRPTTKRVSSQEVGRRQLQCSRCKGIGHNKSTCKEFLLEC
>Mg8
MAKGKLILICQSGGKFVTKGDSTLSYEGGEANAVNIIHETVFDDLKLKVAEMCNLNQKTI
SVKYFLPGNRRNLISLRNDKDLKRMIDFHANSVTADIFVDGEVGFDHDAIKLQASRNSAL
KLAETVNHITAPTTAATPVVNNRKDGADPRVHAHAGSKAAARKVVDSSSPGETYTASPQS
SEHGTDSDSEYKPRVAVSVDADQDLSDLDMTCGPADTVKRRRRTASWTMGARGPTIVAVS
DSDRERRRRKKNNQSREHETDDDILGIDDLGNPSSPGFSDDDLPEKLVASWRDCITGVGQ
DFKSVKEFREALQKYAIAHRFVYKLKKNDSNRASGICVEEGCTWSIHASWVPASLLFRIK
KLNDTHTCGGESWKNAHPAKKLLVSVIKDRLRDSPHDKPREIARSISRDFGIELKYTQVR
RGIEGAREQLQGSYKESYSRLPWFCEKLEETNPGSFVKLLTDDEKRFQCLFVSFLSCVQS
FEKNCRPILFLNATSLKSKYHESLLTATAVDADDGFFPVAFSIVNNENEDNWHWFLEQLK
SALSSSVPLTFVSDRDKGLEKAVHEIFENAHHGYSMYHLIESFKRNLKGPFQGEGRGVLP
GKFLSAAHALRQSVFKKFTEQIKQISPSAYDWVTQVEPEHWTSLSFRGEQYNYIIQNVAE
PYTKLMDEIKESTLMQKIEALIYMISEVINTRRISSSNWTAKLTPSKEKMVQGEALKAHR
LRLFISSDVLFEVHDESTHVVNIEKLECTCLEWKGTSGIPCRHAIAALNSSGKGVYDYCS
KYFTVESYQLTYRESINPIPGIGLPLVKEDAESDDVKVLPPAPRPASEQKKEQSKIEDPD
KRTVTCSKCKEPGHNKASCKATS
>Sb1
MASHELVLGHGQDAELALGPNHHDFGQDHGLGLGHSHELDLGGHAHDHELVLGQSHEHDH
QLVLGHHHDDDHQLVLGHDHHHHGHHVGELALRQGHEGDSGALELEVQGHHHDELDLSES

HELTLAETHHLGVDENLDQLSLEQAHELALQPHDLSQGPLAVAPVVQSRTMVVSPEFQLQ
VGQEFPDVMSCRRAIRNTAIACHFEIQTVKSDKSRFTAKCAAEACPWRIHAAKLPGVPTF
SIRTIHDNHSCVGINHLGHQQASVQWVANTVEERLRENPQCKPKEILEEIHKAHGITLSY
KQAWRGKERIMAAVRGSFEEGYRLLPEYCRQVERTNPGSIARVYGNPDDNCFRRLFISFN
ASIFGFVNACRPLIGLDRTLLKNKYLGTLFLATGFDGDGALFPLAFGVVDEETDENWVWF
LSELHELLEKNTENMPRLTILSDRRKGITDGVEFNFPTAFHGYCMRHVSETFKKEFNNPV
LVNLLWEAAHALTVIEFETKLLEIEDTSSEAVVWIRHLPPRLWATAYFEGTRYGHLTANI
TESLNSWILDASGLPIIQMMECIRRQLMTWFNERREASMQWNTILVPAAERRVQEAIERA
RGYQVARANEAEFEVISAHEGTNIVDIRNRCCLCRGWQLYGVPCAHGVAALLSCRQNVHR
YTESCFTVATYRKTYSQTIHPIPDKTLWNETADHEVADQGQAEETKMEMVINPPKSLRPP
GRPRKKRIRAEDRGRVKRVVHCSRCNQTGHFRTTCAAPI
>Sb2
MEGDQILVQAEQNLIPGAQTLVIGQEFADVDTCRKAIKDMAIALHYELRVVKSDRSRFIA
KCSKEGCPWRVHIAKCPGVPTFTVRTLHGEHKCEGVLNLHHQQATVGWVARSVEARLRDN
PQIKPKEILQDIREQHGVAVSYMQAWRGKERSMAAVNGTLEEGYHFLPAYCGQIVKTNPG
SVATYRGIGPGNAFQRLFVSFRASIYGFLNGCRPLLEIDKADLKGKYLGTLLCASAIDAD
HMMFPLAFGIVDSESDDNWNWFISELRKMLGVNTDKMPVLTILSERKSQVVEAVGSNFPT
AFHGFCLRYVSENFRDEFKNTKLLNLFWSAVYALTPSEFDAKVNEMMQVQDIMPWLQRFP
PNLWAVSYFQGIRYGHFSLGITEILYNLSLDCHELPIVQTIEHIRHQLTCWFAERQNLAQ
SYNSVLVPSAEKLISEAIHDSQCYQVLRANKVEFEVVSSERTNIVDTQARSCSCRRWQIY
GIPCAHAAAALLSCGEDPRLYAHDCFSVMKYRETYSQPIYPIPDRSHWNNSSPGLQGVVS
KADVILSPPNIRRPPGRPKMKILKIESMKRPKRIIQCGRCHLLGHSQKKCSLRI
>Sb3
MARGQILAVLQIGGEFTTDEDGHMSYSGGEAHAMHVQSDWTFSAFKQEISSTLNNLKLDQ
FAFKYFLPKNDKTLISISNDKDLRRMVEFHAESDSTYIYVMKKADNRSKSIVAVSATPTD
AFAISPTTQDGSKRQKVCASWKNVITGVGQVFEGPKDFRDALHKYAIAHRFHYRFIKNDS
SRVTAECTGEGCPWRIHASKSPAKKEFMIKKISESHTCESETVKSNRLASQRWVASVIKE
KLRDSPNYRPRDIANDLQREYGLCLNYSQAWRGRSIAQKELYSTHEEACSQLPWFCERIV
ETNPGSVATVVALEDSKFRFFVAFHASLHGFEHGCRPLLFLEAITAKPNKHWKLLAAASV
DGEGDVFPVAFGVVDDESRENWNWFLEQLKSSLGTSRTITFISNGEHGLWDVVSSVFQES
HHGYCVESLIEEFKTQLDDALTEELKDSMVEHLKKAIYSCTADEFNQYIELIKSESDKLA
DWLLEIKPDQWSDAFFKGSRHGQYSCNVFGTVSEWIPTRYELSVVQLVDTIRCKLMEMMY
TRRESSNAWTEVLTPAANQKLQEEVNKAHTLNVLPAENDENGNMFKVCDDSVNVVNLDTW
ECTCQRWHISGLPCMHAIAVLDRTGQYAYDYCVKYFTTACYRLTYSLSINPIPDVVVPPT
LIDPAQSPATYPCPLRTRRRVGRPKEKPADPRIAIKRAVRCSRCKGYGHNKATCKVPVST
>Sb4
MADGGIVVAICQYGGEFTSGPNGNLTYKGGEAHAVDVSRDMPLESFKDEVSKVFHVDVTD
MSLKYFLPNNYRTLITISCDRDLQRMVDFTISAAQVEVFLISRVENRSVVTYSGASAIKP
GSNAPGDKRKKPPSKNKVSKSNKKTPTPSAPGTSVQPSASNVKQPRPIVTDNDDNRVFQL
EFGSDVAFATPAGGASTAPGVLDQQNLALVDTAPREPIGLFDDSGDPYVGSEITADPQHG
LDNPVMFWDDIIKGVGQEFDNVKDFRAQLCKYAIGKGFAYRFIKNETTRVTVKCVAEGCT
WRLHASESSRNKKFVIKKMTDEHTCGGGNGEGQRRATRQWLTTVIKEKLHDNPMMKPKDL
VKEIYEQYGVTLTYSQVWRGKEVAQKELYHAIRETYSHLPWYCERLTESNPGSIALLSPM
VDTKFRRFFVAFHASLHGFTNGCRPLLFLDKVPLKATNEYKLLVAAAVDADDGVFPVAFS
VVEDENYESWVWFLMQLKFPIQNHSYAYTMTFLSSGQKGLDAAVSQVFGDSHHAFCLHHI
MEEFKGELRKGPWSQQIRDGMIEDFTRAAQACSIEDFNASIESIRNISTEAAEWIIASKP
EHWSDAIFRGCRYDHFSSNIVDAFNNWIPTKKEGSIVLMIDSLRTKIMEIIETRREACKA
WSGPLAPSMEYKAQEEILKAGKMTVLCSSETVFEVRGNAIFVVNLANWECTCRRWQLSGL
PCLHAVAVFNRIGRSFYDYCSKFFRIESYHMTYSGTIFPIPDMDTVDFSAGANLIPPPKP
RTSDKPRRKRLNPNKIPTVIRLCSRCKQAGHNKATCEVIL
>Sb5
MGSRQIVAVLQVGGEFSRDDDGRMSYYGGEAHAMHVKSDWAFKAFKHEISSTLNNLKLDS
YVFKYFLPRNDKTLISISNDKDLKRMVEFHAESETTYIYVMKKADNRLKISIAGADAPAD
CAVIATTPDGSKRQKICASWENAITGAGQVFQSAKEFRDALHKYAIAHRFHYKFVKNDSS

RVTAECTDGGCAWRIHASKSHSKEFMVKKVFGTHTCESETIKSHRLASQKWVASVIKEKI
RDSPNYRPRDIANDLQREYGLCLNYSQAWRGKAIARKELYSSDEEACNQLPWFCQRVVET
NPGSVATVVTLEDSKFRFFVAFHASLHGFEHGCRHLLFLDVISVRSNKHWKLLAATSVDG
EGDIFPVALSVVDDENQENWHWFLEQLNDSLPALGAITFISNGKNGLWDEVSLIFPDSYH
GYHVNCFIEEFKQQLDGSWSEEVKDMMVAHLEKAIYSCKVDEFNQYVELIKTESDKLAEW
LLETKPERWSDAFFKGSRLGQYTCNVSETISDWIPSRYELSVVQLVDTIRCNLMEMMYTR
RESSNTWTEVLTPSANQKLQEQMNKALTLSVVCSTGNDGNGHVFEVCDDSVNVVNIDTWE
CTCRRWHVSGIPCSHAIAVFDRTEQCPLDYCSKYFTTECYRLTYAMSINPIPDVFVPTAA
GDPSQGTTLLSSPILTRRQVGRPKEKPADPRIAIKRAVRCSRCKGYGHNKATCKVPISD
>Sb6
MADGAIVVAICQYGGEFASGPSGNLIYRGGEAHAVDVTRDSSLEGFKDELSKVFHVDVTD
MSLKYFLPNNMKTLITISCDRDLQRMVDFTANAAHVDVFLISRQENRSIVTQSGATSGSV
ASGDKRKRPTSKNKVIRSSKPAASAPGNAVQVITDSLRQPISVTTENEDNRVFQLEFGND
IAFTTTAGGASFTSDILDQQKLALVDNIPREAVSLFDDAFIPYVGSEIMHEPQGPNNPIA
LWDDIIKGVGQEFDNVKDFRAQLCKYAIGKGFVYRFIKNETSRVTVKCAAEGGCTWRLHA
SESSRNKKFVIKRMTDEHTCSGGSGEGQRRATRQWLTTIIKEKLHDNPKFKPKELVKELF
EEYGVTLTYSQVWRGKEVAEKEIYHAIRETRDQLPWYCQRLEETNPGSISVLSPVVDMKY
RRFFVAFHACLHGFVNGCRPLLFLDKVPLKATNDYKLLVAAAIDADDGVFPLAFNVVEDE
NYDSWGWFLMHLKIALQTHNYPCNGMTFLSNGQMGLDAAVSHVFEDGKHAFCLHHIIEEF
KGELRKGPWSLQIREGMVEDFTRAAQACNIEDFNASIESIRNISSEAADWIIASKPEHWS
DAIFRGCRYDNFSLNIADAFDNWIPTKKESSIVLMVDSLRMKIKEIIESRLEACKAWEGP
LTPTMEYKVQDEMLKAGKMTVLCSSETVFEVRGNAIFVVNIGNWECTCRRWQLSGLPCMH
AIAVFNRLGRNLYDYCPKFFRIESYHLTYSGIIFPIPDMDSVDFSAGANLLPLPPKQRSS
DKPRRKRSNSNKISTLVRLCSRCKQTGHNKATCEVQF
