# Supplementary material for: A Gene Family Derived from Transposable Elements during Early Angiosperm Evolution Has Reproductive Fitness Benefits in Arabidopsis thaliana
Source: PLoS Genet. 2012 Sep 6;8(9):e1002931. doi: 10.1371/journal.pgen.1002931 (PMC3435246; doi:10.1371/journal.pgen.1002931)

Figure S2.

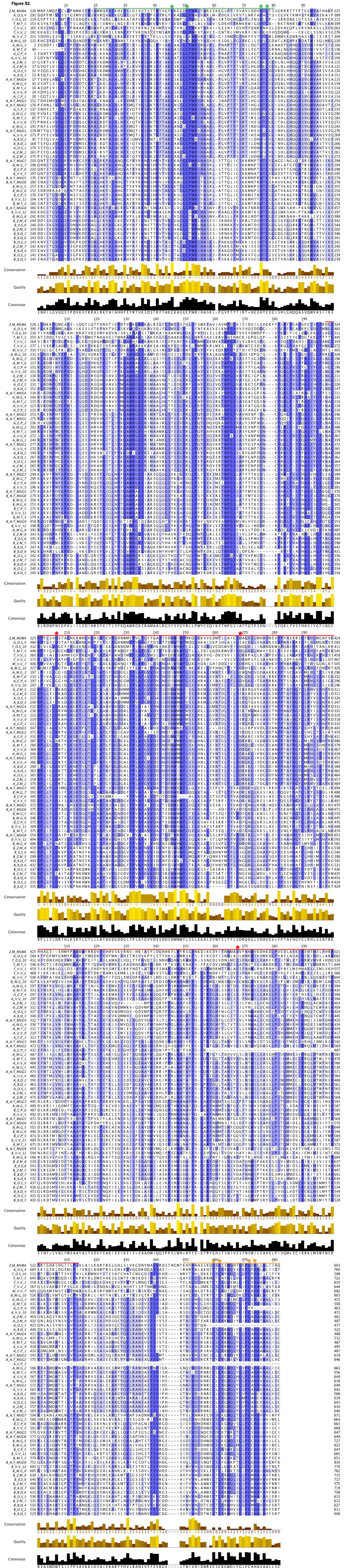

Supplement: Figure S2 — An alignment of MUG and MURA sequences. MUG sequences (A_M.G_10 and below) and MURA of MULEs (M_V.V_2 and above) are shown. Conserved domains are indicated by colored bars and active site residues by asterisks: MuDR (green), MULE (red), SWIM (orange). The CCHC residues of MuDR and CCCH residues of SWIM are conserved in MUG sequences; however, most have mutations to at least one of the DDE residues of the MULE domain. (PDF) [file pgen.1002931.s002.pdf]
