# Supplementary material for: A Gene Family Derived from Transposable Elements during Early Angiosperm Evolution Has Reproductive Fitness Benefits in Arabidopsis thaliana
Source: PLoS Genet. 2012 Sep 6;8(9):e1002931. doi: 10.1371/journal.pgen.1002931 (PMC3435246; doi:10.1371/journal.pgen.1002931)
Supplement: Figure S3 — MUG gene structure showing locations of T-DNA insertions and double mutant allelic combinations. (A) Graphical representation of At-MUG1, At-MUG2, At-MUG7, and At-MUG8 gene transcripts with the position of the two T-DNA insertions for each gene. Bold horizontal lines represent transcripts, dipped lines represent introns, and large blocks represent regions encoding conserved protein domains. (B) Allelic combinations of the double mutants used in the phenotypic analysis. (PDF) [file pgen.1002931.s003.pdf]

Figure S3.

A

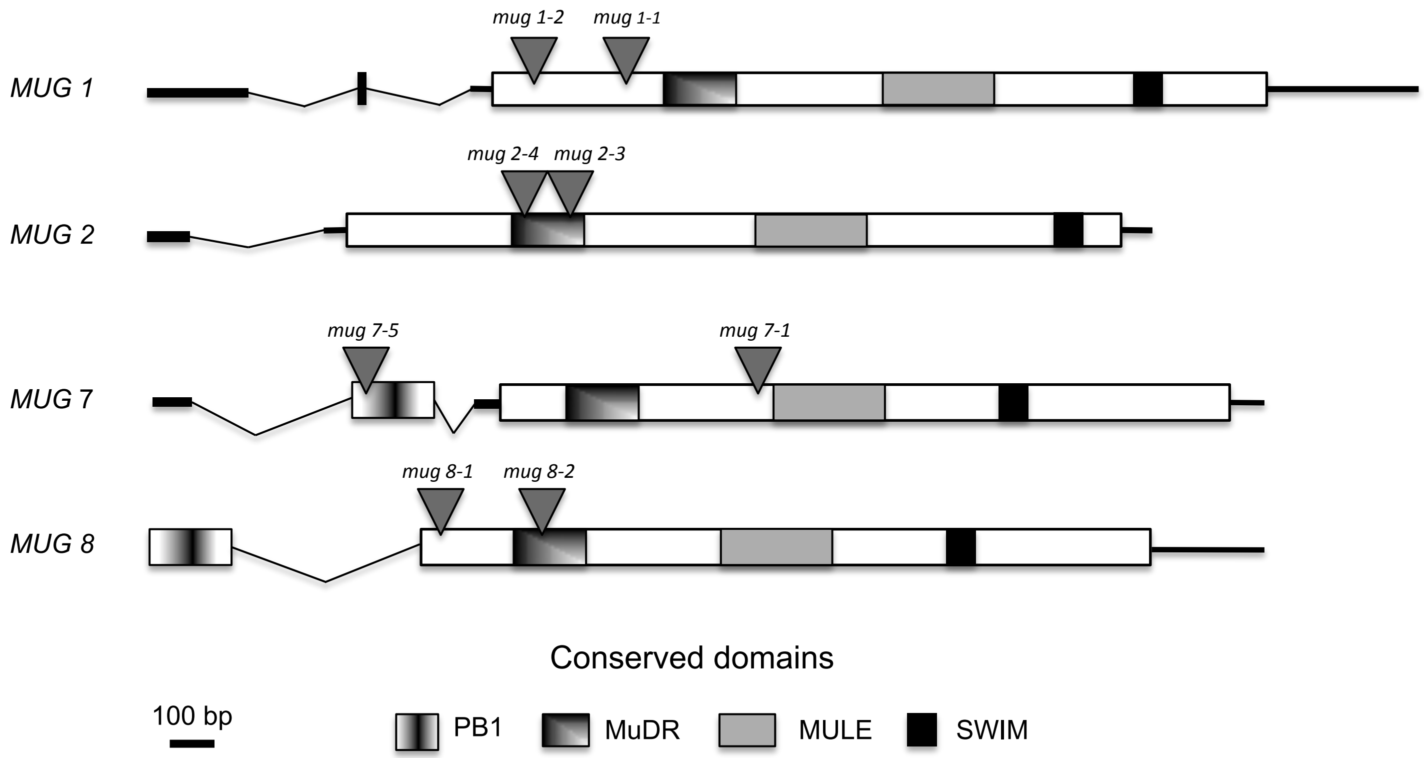

B

**Allelic combination used for *mug1 mug2***	**Allelic combination used for *mug7 mug8***
mug1-1 mug2-3	*mug7-1 mug8-1*
mug1-2 mug2-4	*mug7-5 mug8-2*
