# Supplementary material for: A Gene Family Derived from Transposable Elements during Early Angiosperm Evolution Has Reproductive Fitness Benefits in Arabidopsis thaliana
Source: PLoS Genet. 2012 Sep 6;8(9):e1002931. doi: 10.1371/journal.pgen.1002931 (PMC3435246; doi:10.1371/journal.pgen.1002931)
Supplement: Table S1 — MUG sequences in basal angiosperms. Results of TBLASTN searches of At-MUG1 (MUGA) or At-MUG7 (MUGB) vs. EST consensus sequences from the Ancestral Angiosperm Genome Project (http://ancangio.uga.edu/content/est-assemblies). Counts are estimates based on similarity to At-MUG1 or At-MUG7, the presence or absence of a PB1 domain, the presence or absence of premature stop codons, and phylogenetic analysis. (PDF) [file pgen.1002931.s004.pdf]

Table S1.

Species	MUGA		MUGB		
	# ESTs	Best EST ID	# ESTs	Best EST ID	PB1?
*Zamia furfuracea**	0	-	0	-	-
Amborella trichopoda	6	b4_c19700	0	-	-
Nuphar advena	14	b3_c12933	6	b3_c8318	yes
Aristolochia fimbriata	4	b3_c15276	0	-	-
Liriodendron tulipifera	9	b3_c14809	6	b3_c2867	yes
Persea americana	5	b4_c28181	1	b4_c26687	no

* *Zamia furfuracea* is a cycad, a non-angiosperm seed plant.
