# Supplementary material for: A Gene Family Derived from Transposable Elements during Early Angiosperm Evolution Has Reproductive Fitness Benefits in Arabidopsis thaliana
Source: PLoS Genet. 2012 Sep 6;8(9):e1002931. doi: 10.1371/journal.pgen.1002931 (PMC3435246; doi:10.1371/journal.pgen.1002931)
Supplement: Table S2 — MUG sequences in dbEST. Summary of TBLASTN searches of At-MUG1 (MUGA) or At-MUG7 (MUGB) vs. EST sequences in NCBI dbEST. (PDF) [file pgen.1002931.s005.pdf]

Table S2.

Taxon*	# ESTs in DB	# MUG ESTs
**Tracheophyta, except Euphyllophyta**		
Lycopodiophyta (club-mosses)	101,292	0
**Euphyllophyta, except Spermatophyta**		
Moniliformopses (ferns)	241,806	0
**Spermatophyta, except Angiosperms**		
Coniferophyta (conifers)	1,079,016	0
Cycadophyta (cycads)	42,674	0
Ginkgophyta	21,590	0
Gnetophyta	20,853	0
**Basal Angiosperms**		
Amborellales	26,378	1
Nymphaeales (water lily)	23,686	3
Austrobaileyales	233	0
**Magnoliids**		
Piperales	27,489	2
Laurales	17,425	1
Magnoliales	24,132	6
**Monocots**		
Acorales (sweet flag)	9,695	0
Alismatales	16,059	0
Dioscoreales	44,165	4
Pandanales	1,377	0
Liliales	5,070	2
Asparagales	63,186	7
**Monocots - Commelinids**		
Zingiberales	82,191	4
**Dicots**		
Ranunculales	124,797	21
Proteales	2,238	0
Saxifragales	11,044	1
**Dicots - Rosids**		
Caryophyllales	105,737	6

* Most taxa are orders, except Lycopodiophyta, Coniferophyta, Cycadophyta, Ginkgophyta, and Gnetophyta which are divisions, and Moniliformopses, which is a subdivision.
