# Supplementary material for: A Gene Family Derived from Transposable Elements during Early Angiosperm Evolution Has Reproductive Fitness Benefits in Arabidopsis thaliana
Source: PLoS Genet. 2012 Sep 6;8(9):e1002931. doi: 10.1371/journal.pgen.1002931 (PMC3435246; doi:10.1371/journal.pgen.1002931)
Supplement: Table S3 — Detailed descriptions of fitness metrics. (PDF) [file pgen.1002931.s006.pdf]

Table S3.

Trait	Brief description of measurement method
**% Seed germination**	We recorded the number of seeds that successfully germinated right before transferring them to soil.
**First leaves days after sterilization (DAS)**	We recorded the number of days for each plant to have the development of the first true pair of leaves, not cotyledons.
**Flowering time (DAS)**	We recorded the day at which the first flower fully opened (anthesis).
**Height of main stem inflorescence (cm)**	With a standard ruler, we measured the height of the main stem inflorescence at various time during a period 30 days.
**Diameter of rosette leaves (cm)**	With a standard ruler, we measured the diameter of the longest pair of rosette leaves at various time during a period 30 days.
**Percentage of aborted seeds**	For each genotype, we selected randomly 15 siliques and calculated the proportion of aborted seeds.
**Number of seeds per plant**	For each genotype, we recorded the weight of 100 seeds. Then, we collected all the seeds and weighted the total seed amount. Using the 100 seed ratio, we calculated an approximation of the total number of seeds.
