# Supplementary material for: A Gene Family Derived from Transposable Elements during Early Angiosperm Evolution Has Reproductive Fitness Benefits in Arabidopsis thaliana
Source: PLoS Genet. 2012 Sep 6;8(9):e1002931. doi: 10.1371/journal.pgen.1002931 (PMC3435246; doi:10.1371/journal.pgen.1002931)
Supplement: Table S4 — DDE motifs of MUG proteins. Only variant residues are shown. (PDF) [file pgen.1002931.s007.pdf]

Table S4.

Clade	MUG	D(1)	D(2)	E(3)
A3	Mg3	G	K	-
	Mt6	G	-	-
	Cp4	G	-	-
	Vv10	G	-	-
A2	Zm3	-	E	-
	Zm4	-	E	-
	Sb2	-	E	-
	Os5	-	E	-
	Bd3	-	E	-
	AtMUG4	-	E	-
	Mg4	-	E	-
	Mt2	-	E	-
	Vv9	-	E	-
	Cp1	-	E	-
A1	AtMUG3	-	S	-
	AtMUG2	-	S	-
	Cp3	-	N	-
	Mg2	N	-	G
	Mt1	-	-	-
	Vv6	-	-	-
	Mg1	-	-	-
	AtMUG1	-	-	-
	Vv4	-	-	-
	Bd2	-	-	-
	Bd1	-	-	-
	Os1	-	-	-
	Sb1	-	-	-
	Zm1	-	-	-
	Zm2	-	-	-
Bd2	AtMUG7	-	-	-
	Mg7	-	-	-
	Cp6	-	-	-
	Vv5	-	-	-
Bd1	AtMUG5	-	-	K
	AtMUG6	-	-	L
	Mg5	-	-	Q
	Mg6	-	-	Q
	Cp5	-	-	Q
	Vv11	-	-	Q
	Mt3	-	-	Q
Bd3	AtMUG8	-	-	-
	Vv12	-	-	H
	Mg8	N	-	-
Bm1	Zm8	-	N	D
	Sb6	-	N	D
	Os6	-	S	D
	Zm5	-	S	D
	Sb4	-	S	D
	Bd7	-	N	D
	Bd6	-	N	D
Bm2	Os3	-	N	S
	Zm6	E	N	G
	Sb3	E	N	G
	Bd4	-	N	N
	Os2	-	N	-
	Sb5	-	N	-
	Bd5	-	N	-
