# Supplementary material for: A Gene Family Derived from Transposable Elements during Early Angiosperm Evolution Has Reproductive Fitness Benefits in Arabidopsis thaliana
Source: PLoS Genet. 2012 Sep 6;8(9):e1002931. doi: 10.1371/journal.pgen.1002931 (PMC3435246; doi:10.1371/journal.pgen.1002931)
Supplement: Table S5 — Locus IDs of MUG genes. (PDF) [file pgen.1002931.s008.pdf]

Table S5. MUG loci.

Species	Label	Locus ID* or Location
A. thaliana	AtMUG1	AT3G04605
	AtMUG2	AT2G30640
	AtMUG3	AT1G06740
	AtMUG4	AT5G16505
	AtMUG5	AT3G06940
	AtMUG6	AT5G48965
	AtMUG7	AT3G05850
	AtMUG8	AT5G34853
C. papaya	Cp1	supercontig_123:394,808..396,589
	Cp3	evm.TU.contig_37041
	Cp4	supercontig_20:790,047..791,450
	Cp5	evm.TU.supercontig_146.19
	Cp6	supercontig_48:1,209,999..1,212,321
	Cp7	supercontig_277:54,949..57,928
V. vinifera	Vv4	chr14:8,051,549..8,053,795
	Vv5	chr14:363,591..365,978
	Vv6	chr7:480,947..483,202
	Vv9	chr4:15,764,692..15,766,497
	Vv10	chr3:9,310,218..9,311,966
	Vv11	chr5:2,688,167..2,690,716
	Vv12	chr11:5,970,313..5,972,772
M. guttatus	Mg1	mgv1a002052m.g
	Mg2	mgv1a002269m.g
	Mg3	mgv1a023335m.g
	Mg4	mgv1a006200m.g
	Mg5	mgv1a001707m.g
	Mg6	mgv1a002150m.g
	Mg7	mgv1a001676m.g
	Mg8	mgv1a001259m.g
M. truncatula	Mt1	Medtr1g094000
	Mt2	Medtr7g143350
	Mt3	Medtr8g133990
	Mt6	Medtr4g122010
O. sativa	Os1	LOC_Os12g40530
	Os2	LOC_Os02g09900
	Os3	LOC_Os06g42640
	Os5	LOC_Os01g41210
	Os6	LOC_Os10g01550
B. distachyon	Bd1	Bradi2g19940
	Bd2	Bradi1g23870
	Bd3	Bradi2g02790
	Bd4	Bradi1g35585
	Bd5	Bradi3g06710
	Bd6	Bradi2g03540
	Bd7	Bradi2g36990
Z. mays	Zm1	GRMZM2G423238
	Zm2	GRMZM2G320452
	Zm3	GRMZM2G164709
	Zm4	GRMZM2G363704
	Zm5	GRMZM2G034372
	Zm6	GRMZM2G130905
	Zm8	AC234109.1_FG003
S. bicolor	Sb1	Sb08g020220
	Sb2	Sb03g002010
	Sb3	Sb10g024700
	Sb4	Sb01g027410
	Sb5	Sb04g006220
	Sb6	Sb05g027680

* Locus ID is given wherever a single locus covers most of the MUG sequence (even if the corresponding model does not perfectly match the predicted MUG sequence).
